# Taxonomy of Damage Patterns in Composite Materials, Measuring Signals, and Methods for Automated Damage Diagnostics

**DOI:** 10.3390/ma15134645

**Published:** 2022-07-01

**Authors:** Chirag Shah, Stefan Bosse, Axel von Hehl

**Affiliations:** 1 Chair of Materials Science and Materials Testing (LMW), Faculty IV: School of Science and Technology, Institute for Materials Engineering, University of Siegen, Paul-Bonatz-Straße 9-11, 57076 Siegen, Germany; axel.vhehl@uni-siegen.de; 2 Department of Mathematics and Computer Science, University of Bremen, 28359 Bremen, Germany; sbosse@uni-bremen.de

**Keywords:** composite materials, fibre metal laminates, damage diagnostics, automated feature extraction, machine learning, X-ray computer tomography

## Abstract

Due to the increasing use of the different composite materials in lightweight applications, such as in aerospace, it becomes crucial to understand the different damages occurring within them during life cycle and their possible inspection with different inspection techniques in different life cycle stages. A comprehensive classification of these damage patterns, measuring signals, and analysis methods using a taxonomical approach can help in this direction. In conjunction with the taxonomy, this work addresses damage diagnostics in hybrid and composite materials, such as fibre metal laminates (FMLs). A novel unified taxonomy atlas of damage patterns, measuring signals, and analysis methods is introduced. Analysis methods based on advanced supervised and unsupervised machine learning algorithms, such as autoencoders, self-organising maps, and convolutional neural networks, and a novel z-profiling method, are implemented. Besides formal aspects, an extended use case demonstrating damage identification in FML plates using X-ray computer tomography (X-ray CT) data is used to elaborate different data analysis techniques to amplify or detect damages and to show challenges.

## 1. Introduction

**Figure 1:**
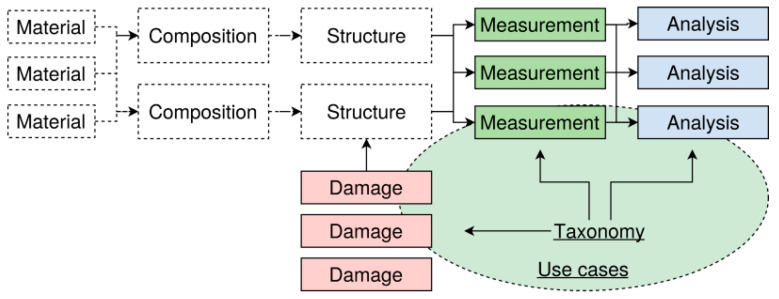
Overview of the work with a focus on the damage taxonomy and damage analysis based on different levels: material, composition, and structure.

Damage diagnostics is still a challenging task requiring export knowledge and advanced analysis algorithms, moreover in the context of hybrid and laminate materials. This work addresses taxonomies of damages, defects, measuring methods, measuring signals, and analysis methods for damage diagnostics of composite and hybrid materials with some selected use cases, shown in Figure 1. The taxonomy is multilevel with material properties on the lowest level, composite characteristics on the midlevel, and structure properties on the highest level. Damages have an impact on the properties at all three levels, which are then measured with different measuring methods. The measuring data are finally analysed by different algorithms.

With the advancements in science, technology, and engineering, there is a vast amount of well-established information available in almost all the respective domains. As research breaks through in these different field progresses, the amount of knowledge and information that is gained also increases. This makes it significantly important to classify the information and the gained knowledge into different well-established classes and in an orderly arrangement. The need for classification has been well realised in various engineering disciplines. The systematic organisation of the investigated subjects helps to organise and structure knowledge in the field of science and technology. Classification helps to facilitate the organisation of existing knowledge. The maturation of various knowledge fields has been facilitated through knowledge classification in the following ways [1]:

- Classification of the knowledge field objects provides common terminology, thus making it easier to share knowledge. The gaps within a knowledge field can be identified through classification [2,3,4].
- A better understanding of the various inter-relationships between the objects of a knowledge field can be achieved through classification, which can support decision-making processes [2].

As the content of knowledge in each discipline increases, further knowledge sharing is becoming increasingly difficult. Disciplines develop different classification systems to facilitate knowledge sharing, which provides a common terminology for communication by allocating entities or subjects to initial undefined classes where the individuals in a class are closely related [3,5]. To create a good classification system, it is crucial to understand its characteristics and what is needed to develop one that can support researchers and practitioners in generalising, communicating, and applying the findings of knowledge upon completion [3]. Classification enables easy identification and makes it easy to understand diversity better. It helps us to understand the relationships between different classes and groups within a subject or domain being examined, further enabling us to uncover and unravel the hidden relationships. It could also provide a significant solution to the storage of a vast amount of data and make the retrieval easier, enabling us to make sense of the world by improving our ability to find important content in an information-rich world [6].

Taxonomy is defined as “a scheme of classification” [7], and the concept was originally proposed by Carolus Linnaeus [8]. In general, it deals with the development of a classification system. Taxonomies have contributed to maturing the knowledge field in various domains. Nevertheless, the taxonomy proposed by Carolus Linnaeus keeps being extended [9], and taxonomies related to materials are expected to evolve over time, incorporating new knowledge and discoveries. In addition, due to the wide spectrum of knowledge available, especially in terms of failure mechanisms and damage patterns in different materials, there is still a need to classify knowledge in many subareas. The detectability and evaluation of these damages are extremely important for damage diagnosis and include numerous analysis methods and techniques. These defects and analysis methods are well known; however, there seems to be a lack of an organisation or classification of these. To the knowledge of the authors, no systematic literature review has been conducted to date to establish a comprehensive classification of these damage patterns, analysis methods, and measuring signals, including their definitions leading to a unified taxonomy atlas. Although many definitions of the damage patterns have been proposed in history, it appears that these definitions have been designed or have evolved without following a proper structure or classification that could explain their origin and occurrences. To the best of our knowledge, no systematic approach or systematic literature review has been conducted to identify, analyse, and classify the different damage patterns in materials. A comprehensive classification and knowledge of these damage patterns, analysis methods, and the corresponding measuring signals could evolve from a better understanding of taxonomies or definitions designed and would be very useful for the development of new taxonomies and the evolution of existing ones. The main contribution of this paper is to develop an approach to create a unified taxonomy for damage diagnosis and for the classification of damages in composite materials using the example of fibre metal laminates (FMLs), followed by an extended use case demonstrating damage detection using X-ray computer tomography (X-ray CT) data to elaborate different data analysis techniques to amplify or detect damages.

We, therefore, attempted to develop a taxonomy method in view of the findings of this study and literature review and from our own experience. The purpose of this paper is to explore the characteristics of damage patterns in materials and their analysis methods, leading to a taxonomy. Here, the authors do not intend to propose or present a final classification method for classifying different damages and analysis methods but to explore the different aspects that could lead to a taxonomy for the classification of different damage patterns occurring within composite and hybrid materials and their detectability using a suitable analysis method for a damage diagnosis.

With this paper, we want to foster a discussion amongst researchers, scientists, engineers, and other practitioners about different damage patterns and their characteristics that could provide the impetus to distinguish different damages and possibly further provide an extension to the identification of these damage patterns with automated damage detection using machine learning methods based on measurement results of non-destructive testing (e.g., X-ray CT, ultrasonic testing).

## 2. Current State of the Art

The importance of taxonomy has been well recognised in recent decades. This section highlights the history of the methodologies implemented and the current developments. 

Various classification structures, such as hierarchy tree and faceted analysis [10], have been used recently to develop taxonomies in knowledge fields, such as education [11], psychology [12], computer science [13], and cyberattack [14]. Taxonomy has also been implemented to manage information and data in maintenance management [15] and for classifying business applications [16] and for smart grid predictive maintenance [17].

There have been numerous applications of taxonomy in recent times, and its implementation is also evident in newly emerging fields of engineering, such as additive manufacturing (AM) [18] and characterisation of engineering design problems [19].

Composite materials, such as fibre-reinforced polymers (FRPs) and fibre metal laminates (FMLs), have wide applications in the aerospace industry due to their high reliability and higher strength-to-weight ratio. The use of these lightweight materials has led to a reduction in the overall weight of aircraft while being in confirmation with the required levels of structural rigidity, further leading to an increased fuel economy. Since these materials have extensive applications in the aerospace and civil aviation industry, the detection and evaluation of the different damage patterns occurring within these become extremely crucial to avoid any possible catastrophic accident. Damages within a composite material can occur at various levels, ranging from damages within the matrix, such as matrix cracks and broken fibres, to failure of laminated elements, resulting in delaminations. The extent of these damages basically determines the residual strength and repeated load life of the material. The detectability and diagnosis of damage patterns are critical to the design of damage-tolerant aerospace structures. Due to a variety of damage patterns occurring within laminates, a comprehensive classification of these damages would enable the structuring of the established knowledge and uniformity in terms of the definitions of these damage patterns.

Besides formal and systematic classification and structuring of damages and their physical patterns, the practical measuring and diagnostics of damages are fundamental. There is a wide range of measuring techniques that can be used to record damage features contained in measuring signals. A damage diagnostics system consists of:

1. A measuring technique;
2. A set of analysis methods;
3. Data;
4. Experts.

Most damage diagnostic applications can be found in structural health monitoring (SHM) systems used for monitoring mechanical structures [20,21] and structural damages [22]. Main measuring techniques are based on mechanical distortion (strain, stress, forces), guided waves (based on ultrasonic waves), and acoustic emission [23]. Testing methods can be classified into destructive and non-destructive techniques. In-depth inspection uses typically X-ray imaging and X-ray tomography [24]. However, detecting damages by visual inspection in composite and hybrid materials is a challenge. For this reason, automated and advanced damage detection methods are required either to aid visual inspection (highlighting regions of interest or damage region candidates) or to perform fully automated damage diagnostics. With respect to the damage pattern taxonomy introduced in this paper, this is, even more, a challenge. Therefore, there are basically the following levels of (automated) damage diagnostics:

1. Detection of a single damage;
2. Classification of the damage;
3. Localisation of a damage;
4. Prediction of the cause of a damage;
5. Prediction of the development of a damage formation.

## 3. Taxonomy of the Damage Patterns in Composite Materials

The first step in the design of a new taxonomy is to clearly define the units of classification. In materials engineering, damages could be classified based on their mode of occurrence, size, location, extent, and so on. For example, damages could be classified as in-process or manufacturing-related defects or could be classified as in-service defects. The initial classes established based on the mode of occurrence could further be subdivided into multiple damage-specific classes, such as impact damages, damages due to environmental influences, damage due to tensile or compressive forces, porosities or voids defects, and defects due to stacking, based on different types. They could then be further classified into sub-sub classes based on their size, location, pattern, and so on. A thorough understanding of the subject matter is required to define clear taxonomy classes that are commonly accepted within a field [25,26]. Once an existing definition of the domain or the subject matter being examined is adopted or is clearly defined, the descriptive terms must also be specified, which can be used to describe and differentiate subject matter instances. To perform a comparison of subject matter instances, an appropriate description of these bases, which can be viewed as a set of attributes that can be used for the classification of the subject matter instances, is important [25,26].

Since taxonomy is a classification of different subjects, it could have multiple approaches, such as a tree or a hierarchy. Taxonomies leading to a single top class that includes all the sub and sub-sub classes (i.e., a hierarchical relationship with inheritances) are known as a hierarchy [10]. For example, consider the hierarchy of students in an institution wherein the top-class student has two subclasses of graduate student and undergraduate student. These subclasses can further be divided into sub-sub classes and so forth. Mutual exclusivity property (i.e., an entity can only belong to one class) is ensured in a true hierarchy, which makes it easier to understand and represent; however, it cannot represent multiple inheritance relationships. In situations where it is necessary to include multiple and diverse criteria for differentiation, the use of hierarchy is not suitable. It is mandatory to define the classes and the differentiating criteria between the classes, including a good knowledge of the subject matter to be classified for hierarchical classification [10].

A tree classification structure is also similar to a hierarchy; however, it has no inheritance relationship between classes, and the common types of relationships between the classes are generally part–whole, cause–effect, and process–product. An example of a tree classification could be a country, its provinces, and its cities [10].

**Figure 2:**
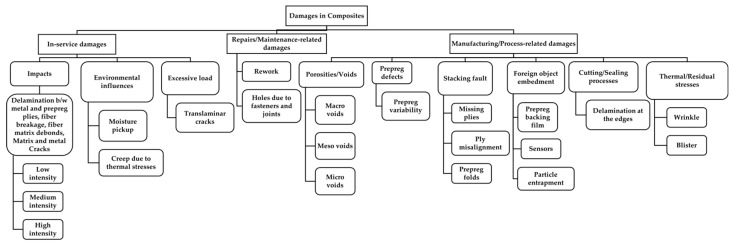
Taxonomy of damage patterns in composites and hybrid materials.

In order to classify different damage patterns occurring within composite materials, the use of hierarchy is more conceivable to highlight all the sub and sub-sub classes, which makes it easier to understand and interpret. The following taxonomy structure has been prepared to accumulate the various kinds of damage patterns occurring under different modes of occurrence (Figure 2). Based on the literature review, the major modes of damage occurrences were found to be in service or during the operation and defects resulting from manufacturing processes. These include damages occurring in civil infrastructures and structures, such as aircraft and wind turbines. Since the proposed classification categorises damages based on their mode of occurrence, here, these have been classified as manufacturing-related damages and in-service damages. Manufacturing damages include anomalies, such as porosity, prepreg defects, defects related to stacking, foreign object embedment, delamination due to the cutting process, and thermal residual stresses resulting from processing discrepancies. They also include such items as inadvertent edge cuts, surface gouges, scratches, pits, and damaged fastener holes due to repairs.

### 3.1. In-Service Damages

#### 3.1.1. Impact Damages

Impact damages include various damage patterns. These patterns include matrix cracking, delamination between the metal and prepreg plies, fibre breakage, metal cracks, and so forth, and are also common to natural fibre composites under impact. The term prepreg refers to a composite material with pre-impregnated bundles of fibres in a polymer matrix (resin) that has been partially cured to be used in a laminate or sandwich structures. These prepreg plies are then directly used in the autoclaving mould for the fabrication of FMLs without the requirement of any additional resin. Damages to components, such as core crush, impact damages, and disbonds, are quite often easy to detect with a visual inspection. Due to their thin face sheets provided, these are significantly larger in extent and are visible with the naked eye. However, there are impact damages that are often difficult to detect with the naked eye as these damages are significantly smaller in size and often do not show any signs of damage on the top and bottom skins of the composite material, thus making them undetectable with a visual inspection. Such damages are often referred to as damages resulting from low-velocity impacts. These damages have internal small delamination, interlaminar debonding, and matrix cracking and could also be accompanied by fibre breakages. Therefore, if these damages are allowed to go unchecked, they could result in the growth of the damage due to liquid or moisture ingression into the core, thus further deteriorating the overall service life of the material [27]. The failure mode of a composite material is basically a two-stage process. Damage is initiated in areas that require low energy consumption, such as the matrix or interface failure, which then continues to a second stage, which requires significantly higher energy, such as fibre breakage. In the first stage, damage begins in areas or regions with lower strength, such as the matrix fibre interface [28]. The interface between the matrix and fibres plays a significant role in stress transfer. For instance, if the fibres are weakly held by the matrix, the composite starts to form a matrix crack at relatively low stress. On the other hand, if the fibres are strongly bonded to the matrix, matrix cracking is delayed, and the composite fails catastrophically because of fibre fracture as the matrix cracks [29]. These matrix cracks could result in intralaminar cracks, which, upon propagation through the prepreg ply, reach the interface between the prepreg layer and the metal ply. At this point, they could give rise to another damage pattern commonly known as delamination, which can be observed in the case of laminated structures. 

**Figure 3:**
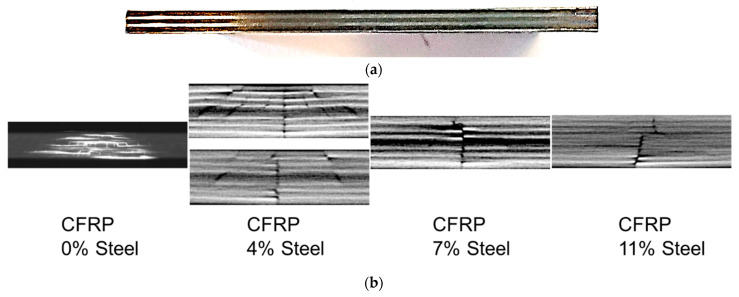
Carbon-fibre-reinforced plastic and steel laminate in a side view (**a**) and typical impact in FML from impact with low energy (**b**).

The further classification of these damages could be challenging as these damages could be present in all the specimens and therefore make a classification based on the presence or absence of these damage patterns difficult. For example, one might think that it is reasonable to classify damage patterns based on various factors, such as impact energy or impact velocities and so on. However, the extent of these damage patterns is indeed based upon the configuration of the specimens. For an instance, two specimens with an identical layup configuration but with different properties of the constituting elements, such as fibre density, prepreg properties, and different layup elements, will experience different damage patterns under identical impact parameters. Due to the differences in damage patterns, these damages cannot be classified into one category or class. For a classification to be based upon impact energies or impact velocities, the material configuration has to be made constant as the extent of damages would differ according to the material configuration. Figure 3 shows that a CFRP–steel (carbon-fibre-reinforced polymer and steel) composite, so-called fibre metal laminate (FML), shows cross-sectional views before after low-energy impacts. Damage patterns with different metal volume fractions are different, although the impact parameters are kept constant.

**Figure 4:**
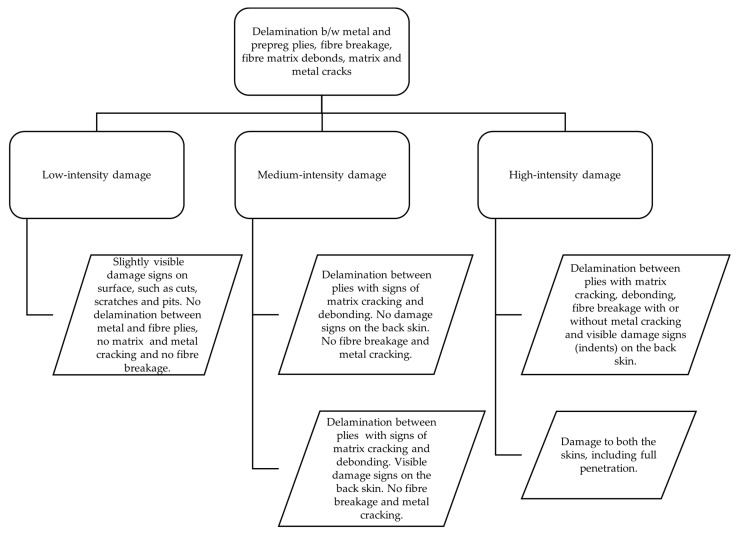
Classification of impact damages based on delamination, fibre breakage, fibre matrix debonds, metal, and matrix cracking.

Considering all the details of different material configurations will increase the complexities for a suitable classification drastically. In order to make the material configuration constant, the volume fraction of the constituting components, which could be besides prepreg, prepreg fibres, and also metal sheets used in FML, has to be made constant. Their volume fraction, orientation, properties, and so on have to be identical for facilitating a classification based on the variety of damage patterns occurring under a variety of impact parameters. This would not lead to a generalised classification scheme, which is applicable to a variety of materials with different configurations under consideration. Damage criteria or damage extent classified as high-energy impact damage would fail to justify the damage patterns occurring in thicker specimens under identical parameters as the difference in layup configurations would lead to different damage patterns. The extent of damages occurring under high-velocity impacts on thicker specimens would be significantly less than that on thinner specimens. For the same impact energy, it was observed that different fibre metal laminate structures showed significantly different damage patterns [30] (as in Figure 3). Classification based on the quantification of these damage patterns is also significantly difficult as the extent of these damage patterns is highly dependent on the material configuration and specifications of the specimen being examined. Hence, it was realised to draw similarities from approaches applied in medicine. In clinical practice, for example, the patient’s illness severity is evaluated on the basis of the assessment of the health conditions (e.g., serious illness or critical illness). The classification of a disease or illness could also be based upon the severity of the symptoms. For example, fever or headache could be classified as mild fever, moderate fever, or severe fever. Each individual classified level could involve various symptoms, which could all be common in between, thus making it significantly difficult to distinguish the different classes from each other based on the presence and absence of these damage elements. To further facilitate the classification, it therefore becomes crucial to distinguish these classes based on the severity of the damages in a similar way to what is being practised in the clinical domain. In the case of impact damages, these therefore could be similarly further classified into three different classes—low-intensity, medium-intensity, and high-intensity damages—as shown in Figure 4.

**Figure 5:**
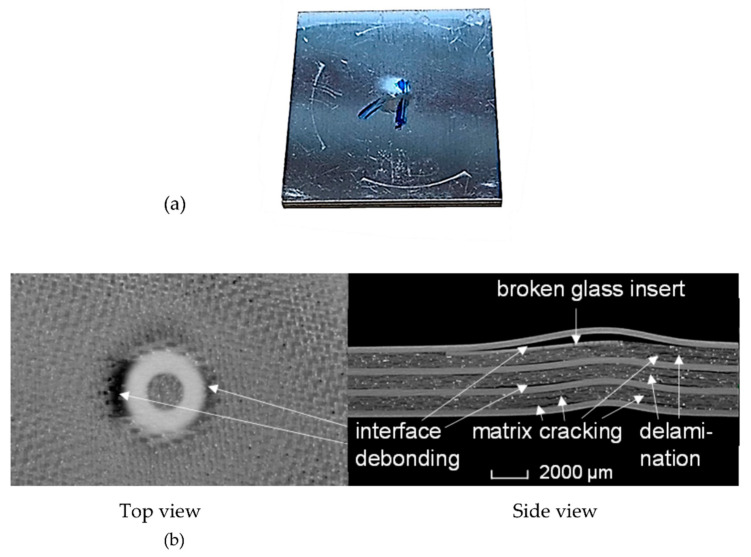
Bottom face of the impacted glass-fibre-reinforced aluminium laminate (GLARE) specimen (**a**) and X-ray CT images of the impact damage through the XY plane (**left**) and cross-sectional view (**right**) (**b**).

Glass-reinforced aluminium, known as GLARE, is a fibre metal laminate (FML) consisting of alternating S2-glass/FM94-epoxy composite plies and 2024-T3 aluminium layers. Panels made of GLARE FMLs are hybrid composites that offer higher damage tolerance characteristics and lower specific mass than monolithic aluminium panels, the reason why they have gained interest from the aerospace industry and have seen widespread applications, especially on the Airbus A380 fuselage and empennage-leading edges [31,32,33]. Figure 5 shows an X-ray CT image of a GLARE 3-4/3 specimen with impact damage acquired using the GE Phoenix v|tome|x M system. Different signs of damage patterns, which include interfacial debonding, matrix cracking, and delamination, are clearly visible. Additionally, a glass insert below the outer aluminium layer of the backside of the FML was used to demonstrate the damage behaviour of an integrated foreign object (e.g., a sensor) during impact.

In addition to the interfaces and fibre rovings, porosities in the epoxy resin can already be observed with X-ray CT. After an impact damage with a hemispherical impactor of 10 mm diameter, a plastic deformed dent with a depth of about 130 µm remains on the impacted aluminium layer. The deformation continues over the entire panel thickness and leads to bending and yielding of the backside aluminium layer and to debonding, especially of the GF–Al interfaces beneath but also in the area of the partially broken glass insert. 

The specimen in Figure 4 can be correlated and classified under the medium intensity damage class with the characteristics of delamination between plies with signs of matrix cracking, debonding, and visible damage on the back skin.

**Figure 6:**
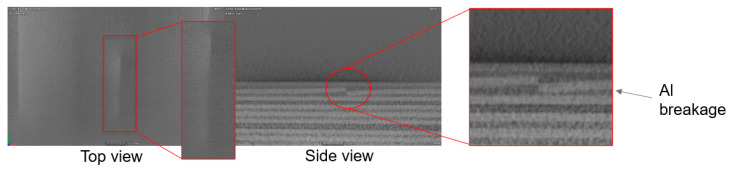
XRCT images of the GLARE specimen with aluminium breakage.

Figure 6 represents a specimen with a crack in one of its constituting aluminium layers. Such metal cracks could be attributed to impacts and excessive loads, leading to intralaminar cracking in the metal layer as, initially, the loads are being carried by the metal layers. Due to this, cracks occur earlier in FMLs than in monolithic materials, such as aluminium. After a fatigue crack has initiated, fibre bridging retards the growth rate, substantially increasing the lifetime to longer cracks compared with monolithic materials. These fatigue cracks can eventually lead to delamination at the interface with fibre layers, which are listed as the most detrimental mechanisms. However, delaminations distribute high stresses over a large area, which allows the bridging fibres to remain intact and contributes to the crack bridging. Epoxies, which are very tough and have high delamination resistance, result in premature fibre failure, further reducing the fatigue life, which has been commonly observed in metals [31]. It has been reported in experimental studies that delaminations only occur at interfaces between plies with different fibre orientations under impact [34].

If two adjacent plies have the same fibre orientation, no delamination will be introduced at the interface between them during impact [34]. 

A GLARE 3-3/2 specimen was fabricated with an artificial fibre breakage. The motive was to replicate the actual fibre breakage occurring in a real-case scenario and identify the detectability of such damages. However, since fibre breakage does not relate to any change in the density of the local damaged region, its detectability with the X-ray CT methods is quite challenging. 

#### 3.1.2. Excessive Loads

**Figure 7:**
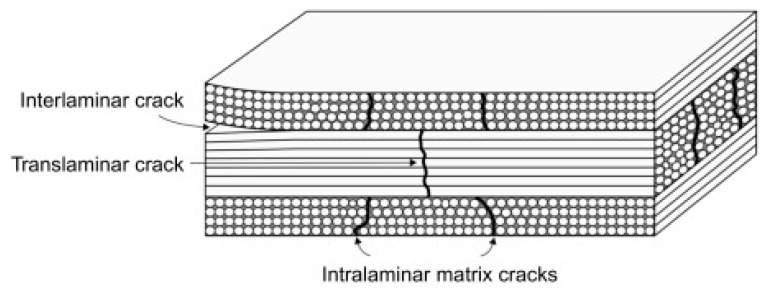
Interlaminar, intralaminar, and translaminar fracture mechanisms [35].

The application of excessive loads could lead to cracking within the laminates. These cracks could be classified as interlaminar, intralaminar and translaminar cracks. Figure 7 shows a schematic diagram of these failure mechanisms.

##### Interlaminar Cracks

Interlaminar cracks are often denoted as delamination, which consists of separation between the constituting plies within a stacked laminate. Delamination can occur at the free edges or cuts or at an exposed surface through the thickness. The laminate develops normal and shear stresses through the thickness at the friction or traction-free surface, extending a short distance into the laminate plane, leading to local cracking in the interlaminar planes [29]. This kind of damage is considered to be the most critical failure mode in a composite material. This is due to the lack of reinforcement fibres in the ‘through thickness’ direction within a composite material; hence, the ply interfaces are the weakest element within it [35]. 

##### Intralaminar Cracks

Intralaminar failure, which is also denoted as ply splits, consists of the formation of in-plane cracks, which are parallel to the reinforcement direction. These cracks may cut across the whole laminate thickness in worst-case scenarios. This usually occurs in unidirectional laminates; however, in multiaxial laminates, ply splits can be effectively bridged by using contiguous plies with different orientations from that where the split initially occurred. These splits, upon propagation through the laminate thickness, reach an interface, which can then lead to delamination, highlighting that there exists some level of interaction between the intralaminar and interlaminar failure, which is highly dependent on the configuration [35]. These cracks can form from defects within a given ply and can grow, traversing the thickness of the ply, running parallel to the fibres in that ply within laminates with different fibre orientations. The very same cracks are invariably referred to as matrix microcracks, transverse cracks, intralaminar cracks, and ply cracks and are caused by tensile loading, fatigue loading, thermal cycling, and changes in temperature [29].

##### Translaminar Cracks

**Figure 8:**
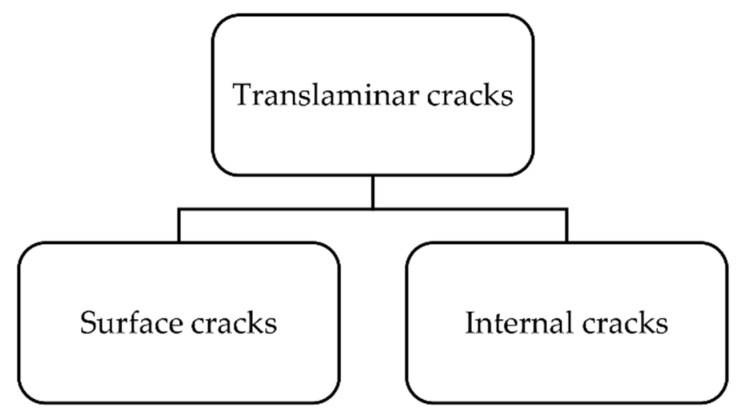
Classification of translaminar cracks.

Translaminar cracks are the ones resulting from the tensile loads and are associated with the tensile or compressive failure of the reinforcement fibres. These cracks are through the thickness cracks where the fibres are broken [27]. The initiation and propagation of translaminar cracks depend on three different failure mechanisms, namely, fibre/matrix debonding, fibre failure, and fibre pull-out, which is directly associated with the fracture toughness. The values of fracture toughness associated with translaminar failure are usually larger (two orders of magnitude) than the value featuring interlaminar cracks. Thus, these translaminar cracks are often neglected in the design process and may still play a role in the failure of notched composite elements [35]. These translaminar cracks could further be divided into surface cracks and internal cracks based on their location within the composite (Figure 8).

#### 3.1.3. Damages Due to Environmental Factors

##### Moisture Absorption

Since composite materials are being extensively used in multiple domains and have numerous structural applications, these materials are often exposed to extreme environments. The environmental effects on such composite materials may pose a threat to the structure and must be taken into consideration during the design process to avoid failure. The susceptibility of these materials to environmental factors depends upon the composition and the configuration of the laminates; therefore, different materials have different sensitivities to environmental factors. The effects of various environmental factors, such as moisture and temperature, can limit the overall performance and deteriorate the mechanical properties during service and the overall usefulness of the material. Such damages are often significant in tropical and subtropical environments, which could cause cracking of the material due to moisture entrapment and could further lead to rapid degradation by corrosion. Water, when absorbed by the matrix within a material, acts as a plasticiser, further softening the material and reducing the properties of the laminates. This moisture upon absorption can migrate along the fibre–matrix interface and could thereby affect the adhesion, reducing the matrix-dominated properties, such as transverse strength, fracture toughness, and impact resistance [36]. It has been reported that with increasing moisture content, the ultimate tensile strength and elastic moduli decrease in 90-degree laminates, which could be as high as 50–90% [37,38]. Hot and humid climate conditions may also affect the performance of the composites and could be another factor responsible for the moisture entrapment. Such defects could lead to a steep reduction in the tensile strength of the material. Excessive and prolonged exposure of the material to harsh climatic conditions could lead to erosion of the resin in areas closer to the surface and could lead to a reduction in service life and could be catastrophic [36]. 

##### Biological Attack

A biological attack consists of a fungal or algal growth. The fungal growth can be attributed to the presence of moisture or wet conditions, which act as a catalyst and lead to marine fouling. However, this fungal growth is occurring at the surfaces, and it does not seem to affect the mechanical properties of the composite and can be removed by scraping. Usually, fungal growth initiates in semimoist conditions and in regions closer to the water–air interface. A repetitive cyclic wetting and drying of the composites can lead to a decrease in the strength of the material [36].

Surface inhibitors and surface chemicals can avoid fungal growth, therefore leading to increased resistance of the material to environmental factors, further increasing the service life.

##### Temperature Effects—Low and High Temperatures and Thermal Stresses

Elevated temperatures for a prolonged period can affect the overall properties of the composite. With an increase in temperature, a loss of stiffness is observed due to matrix softening. The susceptibility of the matrix to softening is dependent not only on the resin but also on the layup. In the presence of moisture, an elevated temperature could also lead to the oxidation of the fibres. Elevated temperature cycles (thermal cycling) between extreme temperatures can also lead to macro- and microcracking within a composite, resulting in a loss of strength [36]. Nanofillers could help combat thermal cracking. The crack bridging effect of the composite material has been found to increase with the addition of nanofillers, such as silicon carbide whiskers, to combat thermomechanical stresses [39].

Overheat conditions generated from a lightning strike could cause catastrophic damage within a laminate. A lightning strike can vaporise the matrix resin and could further create areas of delamination and fibre fracturing in various aircraft components, such as ailerons, composite rudders, wings, and stabiliser tips [36].

Composites and hybrid materials are also subjected to cryogenic temperatures. Due to extremely low temperatures, the composite material starts to behave as a brittle material, leading to a decrease in the shear strength of the material. The temperature effects on the mechanical properties of the composites can also be attributed to the different thermal expansion coefficients of the constituting elements, leading to internal stresses. These internal stresses change their magnitude with changes in temperature, producing matrix cracking at very low temperatures in some cases.

A polymer used within a laminate has an operating temperature limit, which is slightly below its glass transition temperature, where the polymer transits from a glasslike substance to a rubbery state and suffers a substantial reduction in mechanical properties [36]. The temperature effect on the fibre–matrix interface as strong as that of the fibre treatment and resin properties has been reported [40].

### 3.2. Repairs/Maintenance-Related Damages

Repairs and maintenance-related damages are damages occurring during repairs. These mainly account for fastener holes, reworked areas, and other surface damages, such as surface marks due to surface treatments using specialised chemicals and so on.

### 3.3. Manufacturing/Process-Related Damages 

#### 3.3.1. Foreign Object Embedment

**Figure 9:**
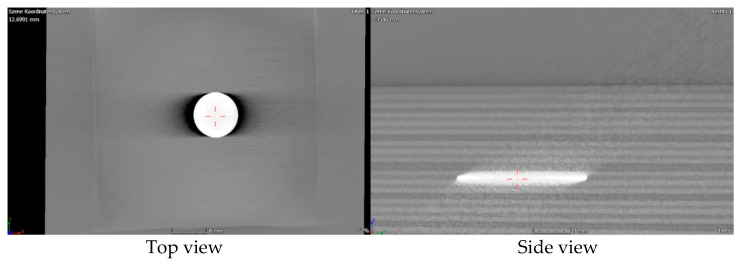
XRCT images of a GLARE specimen with an embedded dummy sensor.

**Figure 10:**
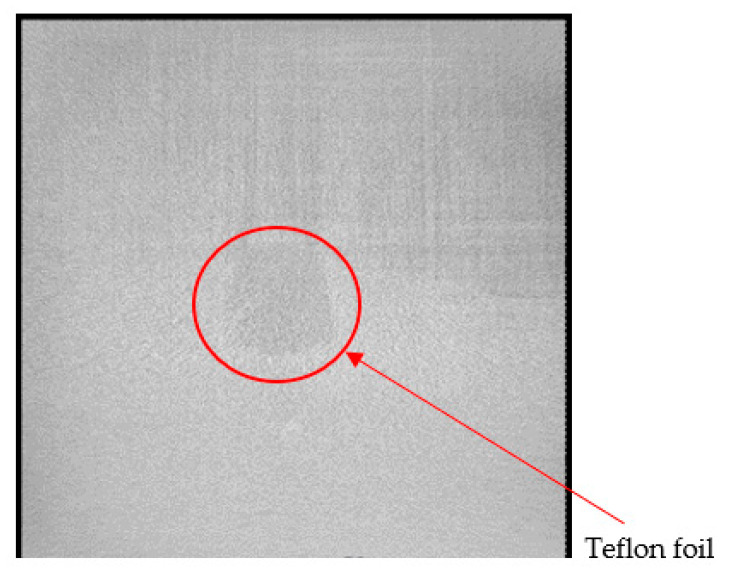
GLARE specimen with an integrated Teflon foil.

**Figure 11:**
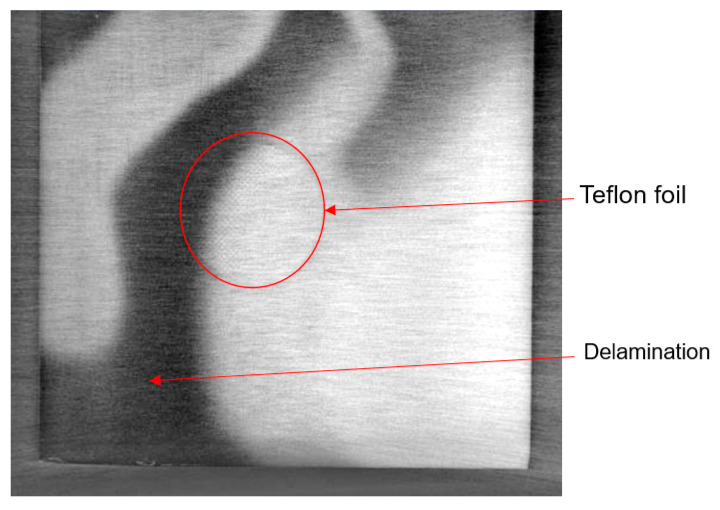
XRCT images of a GLARE specimen with an embedded Teflon foil, resulting in delamination in the laminate.

To our knowledge, foreign object embedment refers to an embedment of a component that does not constitute the laminate or composite and has a different material composition than that of the composite or laminate. For structural health monitoring purposes, composite materials can also be fabricated with an integrated sensor network. A micro-oscillator as an integrable sensor for structure-borne ultrasound has been investigated, where the sensor response has been discussed in regard to its usability for SHM [41]. Inefficient integration of these sensors could lead to delamination and debonding between different plies. It could also lead to a lack of adhesion in localised areas where the sensors are located, thus initiating a local site for damage propagation. Figure 9 shows an X-ray CT image of a specimen with an integrated dummy sensor. This sensor was identified in the image due to the difference in density in comparison with the laminate’s constituting metal and prepreg plies. Prepregs are often covered with a transparent backing film at the bottom to protect them from environmental influences or contamination. Improper removal of this film could lead to an embedment of the film into the composite, leading to inhomogeneities and even delamination, which could be catastrophic and pose a serious problem. Figure 10 and Figure 11 show X-ray CT images of specimens with artificially created delamination using a Teflon tape. These specimens were fabricated by embedding a Teflon tape into the laminate to understand its detectability using X-ray CT methods. In Figure 11, delamination in the dark region can be seen, which can be attributed to the embedment of the Teflon tape. This tape resulted in a lack of adhesion in between the layers, thus resulting in delamination at the edges, which intensified during the cutting process due to thermomechanical stresses. These delaminations further lead to the entrapment of the metal fragments resulting from the cutting process.

Improper handling could account for the embedment of a backing film and could also leave greasy finger marks on the surface of the prepreg, which could accompany dirt and so on. Common examples of manufacturing-related flaws include a contaminated bonding surface or inclusions, such as a prepreg separation film or a backing paper that is inadvertently left between plies during layup [27].

##### Particle Entrapment

**Figure 12:**
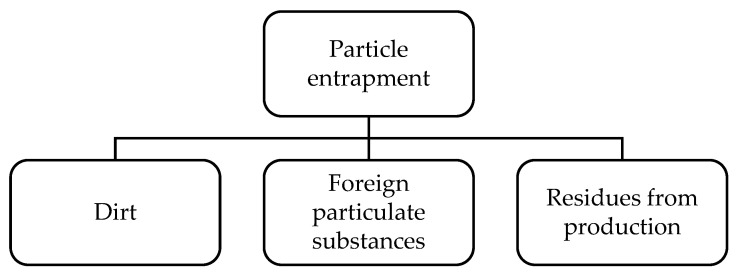
Classification of particle entrapment in composite materials.

To the authors’ understanding, particle entrapment is another type of manufacturing-related defect that occurs within the composite materials during the manufacturing process. It is mainly a fabrication-related defect arising due to poor standardisation of the fabrication process or techniques and often occurs during the fabrication of the component. In general, particle entrapment could include various elements, such as dirt or any foreign particulate substances getting embedded into the composite due to the fabrication in a dusty or unfavourable environment, which could also include residues from the production process (Figure 12). Such foreign particle inclusions are considered to be contaminants and can pose a serious risk on the structural integrity of the specimen and deteriorate the overall mechanical performance.

Such defects could also arise from the lack of training or fabrication skills of the technicians in the fabrication department. Improper cutting processes could also lead to the entrapment of the metal particles into the composite, leading to internal contamination. The inclusion of foreign bodies in the matrix is another defect that happens during the manufacturing process of the prepreg plies, which range from dust to metal particles and other similar contaminants. Inadvertent (nonprocess) damage can occur in parts or components during assembly or transport or during operation.

**Figure 13:**
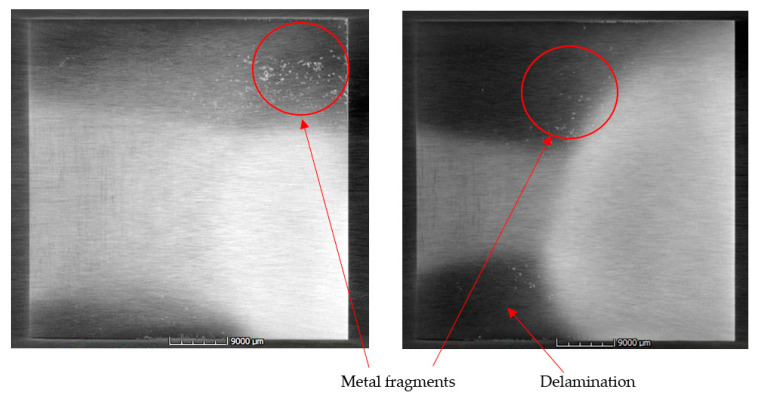
X-ray CT images of a GLARE specimen with metal fragments from the cutting process embedded into the laminate.

The laminate cutting process could also result in some delamination around the edges. This is due to the high thermal stress acting on the edges of the laminate during the cutting operation. During this process, tiny metal particles or metal fragments resulting from the process could get into the laminate itself, which could be detrimental to the overall structure. Figure 13 shows X-ray CT images of the laminate with metal fragments getting embedded into the laminate.

Human error is also a contributing factor to manufacturing defects. From the authors’ experience, manufacturing defects can also account for an improper stacking of the constituting plies within a laminate, which is a result of human error. This could result in a faulty layup configuration of the laminate or improper orientation of the plies. Human error could also account for missing plies within a laminate.

Other sources of manufacturing defects can also include improper machining, using substandard material, inadequate tooling, mishandling, and mislocation of holes or details [27].

#### 3.3.2. Thermal/Residual Stresses

##### Wrinkle

The effect of manufacturing on polymer composites resulting in decreased mechanical performance is basically due to ply/fibre waviness or wrinkling conditions. Fibre waviness is known as a fibre deviation from a straight alignment and/or as a wave-formed ply in a unidirectional laminate, which might be due to the detrimental manufacturing effect that generally occurs during consolidation/curing and infiltration and/or as a result of the draping process. Fibre buckling or buckles are referred to as out-of-plane fibre waviness, which happens due to stability issues when the ply undergoes compression loading [42]. For monolayer composites, the fabric deforms in and out of the plane, taking the shape of a waved curve that can vary in length, number, and magnitude; such defects are known as wrinkles. For multilayer materials, different radii between the inner layers and the outer layers at the corner result in an inner layer buckling to cope with the compressive force [43]. This gives rise to another pattern of wrinkles that are seen in multilayer materials. The properties of the wrinkles, such as number and magnitude, depend on the bending stiffness of the fabric, where the magnitude of these wrinkles increases along with the bending stiffness, which has also been proven [44,45].

Thermal effects during curing (differential thermal contraction) can lead to the development of surface wrinkles, particularly if the layers are thin and lack out-of-plane support. These surface wrinkles are of particular concern under bending, buckling, or compression, as there is no lateral support on the wrinkled layers in these instances, and therefore, fibre buckling is further promoted [46].

##### Blister

**Figure 14:**
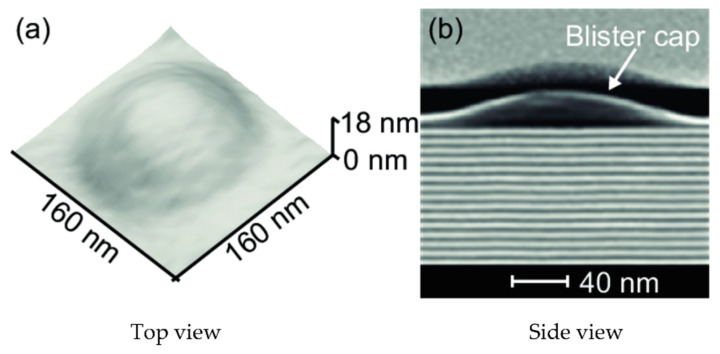
Blisters within a laminate: (**a**) blister on the surface; (**b**) blister inside the laminate [27].

Blisters are another damage pattern commonly seen within laminates. These are regions in the laminate that are identified by plies deforming out of the plane from the laminate and are caused by the expansion of trapped gases within the laminate (Figure 14). These could occur due to chemical attacks or localised heating of the matrix [27]. Moisture trapped within a composite material during rapid heating can result in plasticisation, hydrolysis, and blistering [47].

The initiation of a blister depends on four principal criteria, which include steam pressure, resin stress–strain behaviour, temperature, and initiation sites. Blistering occurs when there is excessive moisture in the resin and when the temperature is high enough for the moisture pressure to exceed the strength of the material. It follows that a temperature–moisture concentration envelope may be developed that delineates conditions under which blistering would not occur for the safe operation of the part [48].

#### 3.3.3. Stacking Fault

##### Prepreg Folds

After the formation of the wrinkle curves, the application of the normal forces on the stack of layers can result in these curve sides collapsing on the other, further multiplying the thickness of the local area depending on the number of layers folded [45]. This leads to a local increase in fibre volume fraction at the defect location in areas where these folds are present. Within each prepreg fold, two additional plies are added over the laminate thickness [49].

**Figure 15:**
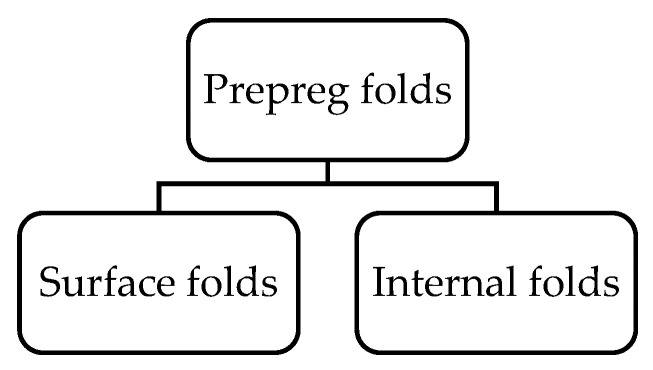
Classification of prepreg folds.

Based on the location of the prepreg folds, these folds could further be classified as surface folds and internal folds (Figure 15). Surface folds are the prepreg folds occurring in the layers closer to the surface of the laminate, whereas the folds inside can be classified as internal folds.

#### 3.3.4. Prepreg Defects

##### Prepreg Variability

**Figure 16:**
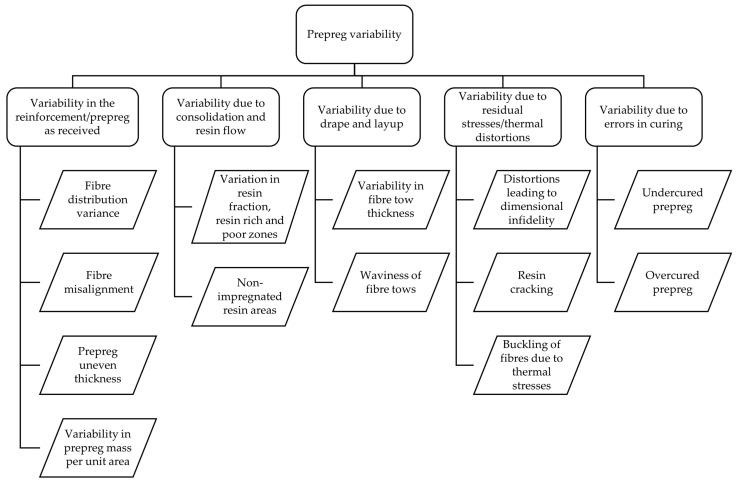
Classification of prepreg variability in composite materials.

Prepreg variability can be attributed to multiple reasons originating from the manufacturing or fabrication cycles, or it could also be due to various in-service factors, such as thermal stresses, drape or layup over a surface, and so forth. A comprehensive classification of different damages resulting from prepreg variability is shown below in Figure 16.

##### Variability in the Reinforcement/Prepreg as Received

Variability in the prepreg reinforcement could also lead to non-conformance with the required prepreg standard. Incoming materials can have variability and have a significant and direct impact on the quality in terms of mass/unit area and, hence, the ply and laminate thickness. It may also have a direct impact on void content provided that the process design does not account for the variability of the incoming materials [50].

Variability in terms of mass/unit area of the prepreg can also be noted especially in the prepreg stored on a roller drum. Due to this, prepregs are often accompanied by a tendency to have a higher mass/unit area on one side of the roll than the other, which is probably associated with the stiffness and alignment of the rollers used in the prepreg process [50]. Fibre misalignment within a prepreg could also lead to localised regions with high-stress concentrations, which could lead to a complete failure. These misalignments can most likely be attributed to the wrapping of the prepreg onto a storage drum. As the path length on the outside of the drum is longer than that on the inside surface, the fibres on the inside must buckle to accommodate these path differences, thus leading to wrinkles. As the prepreg is unwrapped and flattened, the extremely viscoelastic nature of the prepreg makes it difficult for these wrinkles to be fully and immediately relieved, therefore making it reasonable to assume that the fibre waviness seen in a flat prepreg can be largely attributed to the rolling of the prepreg onto a storage drum after the manufacturing process [50].

##### Variability Due to Consolidation and Resin Flow

Resin-rich zones causing unwanted residual stress, deformation, and part-to-part variation are regarded as the most common phenomenon in the liquid composite moulding process [51,52,53].

**Figure 17:**
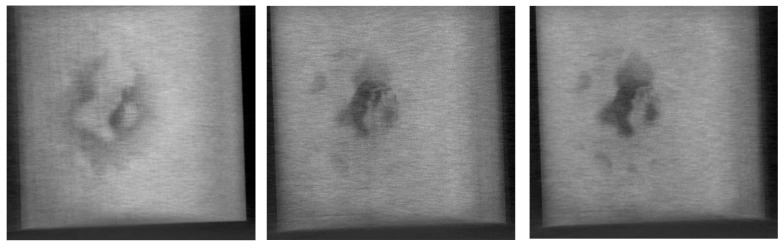
Glare specimen with nonuniform resin regions.

The resin-rich zones are formed during resin transfer in the moulding process [54]. A part is referred to as resin rich if too much resin is used. This adds weight to the composite. A part is called resin starved if insufficient resin is applied during the wet layup process or too much resin has bled off during the curing process. Such areas are indicated by fibres showing on the surface and could lead to regions with nonimpregnated resin, which could cause fibre misalignment and, as a result, significantly reduce the strength. A 60:40 fibre-to-resin ratio is considered optimum [27]. Resin insufficiency or oversufficiency could also lead to porosities or voids within the material. Figure 17 shows X-ray CT images of specimens with inhomogeneities in the prepreg resin fractions. The visible dark patches in the images are the regions with resin being washed out using an acetone solution.

##### Variability Due to Drape and Layup

The layup of the prepreg or the polymers with embedded fibres can result in different defects arising from the draping of the composite onto a particular surface. If the tows or bundles of the nominally straight fibres are draped over a surface with a simple radius, the fibres on the inside and on the side closer to the surface will experience different loading conditions [50]. The fibres on the outside will undergo tensile loading, and the fibres on the inside are loaded in compression. This leads to the buckling of the fibres. This makes it extremely critical to figure out the right geometry for the fabrication, which could lead to a lower buckling of these fibres.

##### Variability Due to Residual Stresses/Thermal Distortion

Thermal stresses and excursions associated with the curing processes lead to internal residual stresses in all the composite components [50]. A balanced set of stresses in the fibres and matrix is crucial for the overall integrity of the specimen. The fibres and matrix have different thermal expansion coefficients, which could lead to stresses within a matrix itself and could initiate fibre–matrix disbands. The coefficients parallel and perpendicular to the fibre will lead to stresses between different plies with different alignments [50]. Non-homogeneity of the resin consistency and nonuniformity of the resin zones would worsen the effects of residual and thermal stresses. Since a laminate consists of different constituting elements comprising metal and prepreg plies, differences in stresses through the thickness are also evident, leading to thermoelastic distortion resulting from differences in in-plane and through-thickness thermal expansion coefficients.

#### 3.3.5. Porosity and Voids

Void formation has several causes, such as mechanical air entrapment during resin flow, which is identified as the main cause [55]; gas created during the chemical reactions in the curing cycles [56], and nucleation of dissolved gases within the resin [57]. The inhomogeneous fibre architecture results in nonuniform permeability of the fibre preform, causing local variation in the resin velocity, leading to air entrapment. The capillary effect prevailing at the microscale exacerbates the local velocity [58].

Voids are formed at three different scales: macro, meso, and micro. Voids in between the fibres in a bundle or a tow are referred to as micro-voids, in between the tows as meso-voids, and in larger zone of the preform (visible to the naked eye) as macro-voids. Microscale flow at the tow level relating to the heterogeneous medium of the preform controls the micro- and meso-void formation, whereas macroscopic or global flow considering the preform as a homogeneous medium dictates the formation of the macro-voids. The macroscopic and microscale flows interact with each other and are strongly coupled [59,60].

**Figure 18:**
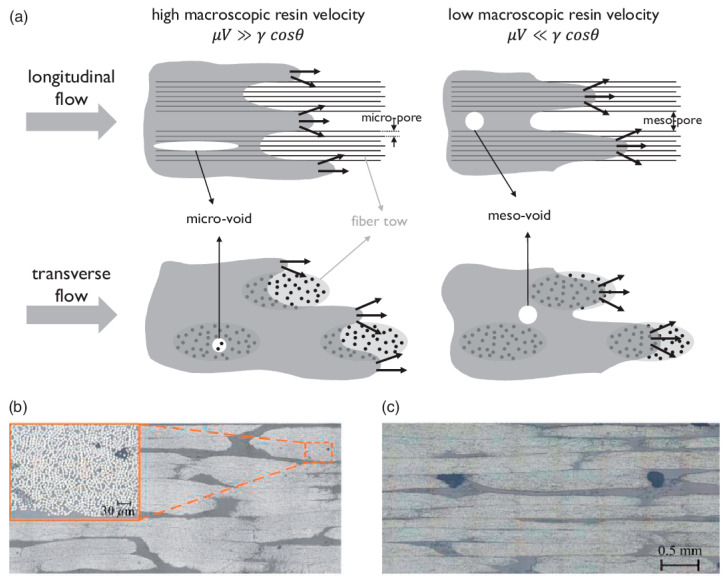
(**a**) Schematic of void formation during longitudinal and transverse flow in liquid composite moulding of a dual-scale fibrous preform, exhibiting a competition between the viscous flow and the capillary flow—inclined arrows show the transverse impregnation of the tow; micrographs showing [60] (**b**) micro- and (**c**) meso-voids inside and between tows, respectively [61].

Voids have been called differently in the literature. For instance, a macro-void is known “dry spot”; a meso-void as “interbundle”, “intertow”, or “channel” void; and a micro-void as “intrabundle”, “intratow”, or “tow” void [59]. Figure 18 shows a schematic of void formation in longitudinal and transverse flows in liquid composite moulding.

## 4. Taxonomy of Features

Damage diagnostics and prediction are a complex system that consists of different levels:

1. Measuring principle and physical interaction mechanisms;
2. Sensor and signal measurement: physical variable → signal;
3. Sensor data calibration;
4. Signal preprocessing;
5. Sensor data normalisation;
6. Data reduction;
7. Damage detection and localisation;
8. Damage characterisation.

Therefore, there are different features:

- DMF: Damage pattern features (as introduced in the previous sections) characterising damages from the material science of view;
- BSF: Basic signal features (e.g., statistical features);
- IF: Intermediate features (e.g., frequency spectrum), signal codings, ROI markings, local-point damage detection;
- SFD: Damage features in the signal; that is, any deviation of a signal from a baseline (no damage) signal is a signal-damage feature (in time, spatial, or frequency domain), typically low level and still ambiguous;
- OF: Output features of a (automated) diagnostic system, that is, damage features referencing some subset of the DMF set;
- GOF: Geometrical features as a subset (e.g., damage position, damage shape).

Features as an output from a damage detector function are typically only indicators that are weakly correlated to the damage patterns as a result of ambiguity, uncertainty, and specialised detection models. For example, a binary output from a damage detector can cover a broad range of damages and cannot distinguish between, for example, resin defects and impact damages with delaminations.

## 5. Taxonomy of Measuring Signals and Methods in Composite Materials

There are three basic output features in the damage detection process: the existence, location, and extent of the damage. Measuring signals are commonly used to discriminate and compute these basic damage features. In addition to the consideration of the basic classification of damages in composites, the automated detectability of the various damages and defects by metrological methods is of great importance. Therefore, a classification of various mathematical properties of measurement signals will be considered in this section. Finally, the analysis methods with which damage and defect characteristics can be inferred from the measurement signals are considered in the following section. Input and output features must be distinguished. The input features are essential properties of the measurement signal (the input variables *x*), which make it possible to infer the output features (the output variables *y*), that is, the damage and defect features or any related information in the temporal and spatial domain.

There is a measuring signal *s_m_* delivered by a specific measuring method *m* that is a result of and depends on the physical interaction of a specimen under test *d*, some kind of excitation signal *s_e_*, and the environment *E* (not further characterised). The measuring signal can be time dependent and is always a composition of a function of the physical variables *f*(*v_x_*) to be measured and a noise signal *g*(*v_n_*). The physical variable can be of primary interest (e.g., temperature) or of secondary interest. The activation *s_a_* can be injected explicitly only for a specific measurement by an actuator or already be existing by environmental excitations, for example, acoustic waves as a result of machine operation. Some physical variables are statistical aggregates (e.g., material temperature, moisture, and pressure). These variables can be measured directly without excitation.

There is a known or unknown model *M* that defines the relationship of the excitation signal *s_e_* and the resulting measuring signal *s_m_* with some parameters *p* (e.g., damages, defects, or other environmental variables).

(1) $$\begin{array}{l} s_{m}=s_{m}\left(v_{x},v_{n},E,t\right)=f\left(v_{x}\right)+g\left(v_{n}\right)\\ M\left(m,d,p,E\right):s_{e}\to s_{m} \end{array}$$

**Figure 19:**
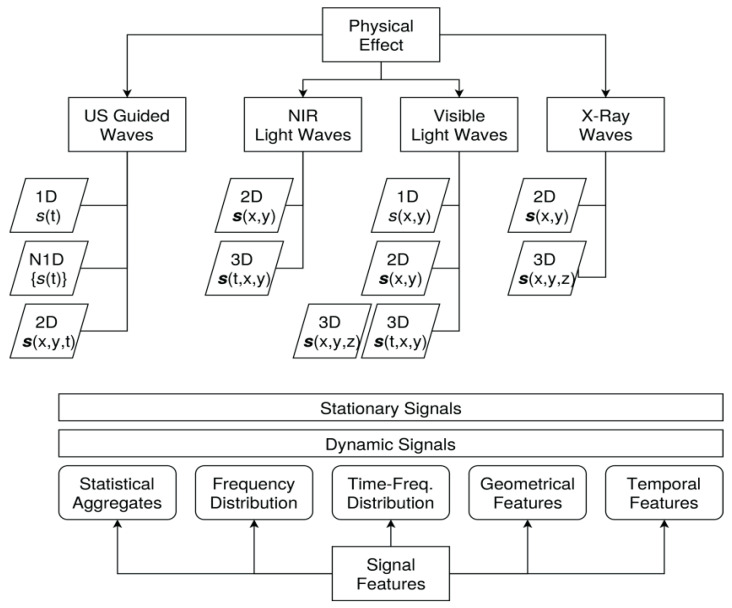
(Top) Coarse overview of different measuring signal classes with respect to their physical interaction effects. (Bottom) Common derived signal features relevant for damage diagnostics. Shown are the dimensionality of the data and the dependent variables (US: ultrasonic; NIR: near-infrared).

Most physical effects in damage diagnostics are related to wave interaction (e.g., guided ultrasonic waves (GUW) or light and X-ray waves). The measuring signal is dependent on the wave interaction with the specimen material and any kind of damage. This means that the signal *s_m_* contains material and damage-relevant features in the time or spatial domain. Common signal classes with respect to physical effects and measuring techniques are summarised in Figure 19. There are basically two classes of signals in the time domain and four classes in the spatial domain (dimensionality) that are important for the application of the following analysis and diagnostic methods:

1. Scalar and time-independent (stationary) signal variables *s* (e.g., temperature or light intensity);
2. Vectorial time-independent (stationary) signal variables *s* (e.g., strain or stress);
3. Two-dimensional time-dependent (stationary) matrix signal variables *s* = *s*(*x*,*y*) (e.g., an X-ray or light image);
4. Three-dimensional time-dependent (stationary) matrix or tensor signal variables *s* = *s*(*x*,*y*,*z*);
5. Time-dependent signals of classes 1–4 (i.e., *s* = *s*(*t*)).

Typical examples of measuring techniques used in damage diagnostics are:

**Guided Ultrasonic Waves**: Using ultrasonic signals with active excitation, damages modify wave propagation; signals are time-resolved vectors of real or complex values, as a response of the excitation signal;
**Light Microscopy**: Using visible, near-infrared, or polarised light, signals are 2D real-value images; damages modify the surface structure of material slices;
**Interferometry**: Using laser light, signals are 2D real- or complex-value images; damages modify surface geometry;
**X-ray Imaging**: In-depth imaging (typically in transmission mode) of materials using X-ray waves in the energy range of 30–100 keV; signals are 2D real-value images or 1D z-profiles;
**X-ray Tomography**: Signals are 3D real-value images.

Sensor signals from a measurement contain information about damages (signal-damage features) that must be typically extracted by using numerical, analytical, statistical, or ML methods. With respect to signal images, there are different geometrical features to be distinguished, basically dividing the feature space into one-, two- and three-dimensional feature objects (1D, 2D, 3D):

1. Single or a few spatially extended but spatially limited and larger feature regions with a characterised shape, for example, a circle or a triangle as a result of localised damages;
2. Single or a few spatially extended but without a characteristic shape, as a result, for example, from layer delamination;
3. Multiple smaller spatially extended feature regions not clearly bound;
4. Single straight and extended lines or rectangles with a high width-to-height ratio, characterised by length, width, and angle, as a result of, for example, cracks or fibre breakages;
5. Multiple shorter lines or rectangles with a high width-to-height ratio, as a result of, for example, cracks or fibre breakages;
6. Speckles, that is, small statistically distributed spots without a specific geometry; and finally,
7. Noise.

In a damage diagnostics system, there are physical sensor variables that are characterised by an immediate result of a measurement by measuring a physical variable. Besides physical sensors, there are virtual sensors that are aggregates or transformations of physical sensors or other virtual sensors, creating intermediate features that are correlated with damage features. Examples are statistical aggregates and transformations, for example, from time to frequency space. These transformations can create stationary variables from dynamic (time-dependent) variables. Furthermore, transformation can create “translation and rotation” invariant variables. For example, a time-dependent signal can be characterised by a dedicated but possibly unknown start and end time, important for some damage diagnostic methods. After time-to-frequency transformation, this feature is removed (and an additional constant signal offset), and the signal is normalised. Signal offsets can be removed by high-pass filters or gradient transformations.

In the following sections and the use-case section, measured signal data are handled always as multidimensional data volumes. Each element of a data volume *V* is a scalar value.

## 6. Taxonomy of Analysis Methods

A single measurement of a signal *s_m_* is considered one experiment and represents one row *r* in the data table *D* that is used to derive damage features from the measurements of a specimen under test, for example, in a destructive or non-destructive mechanical test.

The analysis methods can be basically classified in:

1. Analytical methods based on physical, material, or structural models used for modelling the relationship between the activation signal, the measuring signal, and the damage;
2. Statistical methods;
3. Regression methods;
4. Classification methods;
5. Correlation methods (clustering).

Learning of damage feature extraction models from data is mostly a minimisation problem fitting a model to a given data set (data-driven modelling).

Analysis (i.e., damage diagnostics) methods can be applied to the entire sensor data set (global context), to parts of the sensor data set (segment context), or to small extracted pieces (local context). An important aspect in damage diagnostics is the invariance with respect to translation and rotation, simply viewed in a geometrical context. For example, a time shift or offset of a measuring signal may have no influence on the output feature detection. Considering images, a damage classifier must be insensitive to the location of the damage signal feature contained in the image. Previous transformations can reduce or eliminate the effect of translation or rotation in time and geometrical space, for example, by using time-to-frequency transformations, such as the Fourier transform. However, any signal transformation typically results in a loss of information.

### 6.1. Statistical Methods

It is assumed that there is a measuring signal *s*(*i*) that consists of a series of single values *s_i_* for *i* = 1, 2, …, *N*. Statistical methods can deliver signal aggregate feature variables that are related to damages and defects. Common statistical aggregate variables are:

- Mean s_, minimum, and maximum signal values smin and smax and their positions imin and imax;
- Standard deviation of signal value distribution:
- Skewness of signal value distribution;
- Correlation coefficients between two variables (data and time series) or autocorrelation of one variable;
- Signal energy *E*(*s*) of the L1 norm (area of the signal relative to another curve or line *g*(*i*), a constant *g* = const):

(2) $$\begin{array}{l} E_{L1}\left(s\right)=\frac{1}{N}\sum_{i=1}^{N}\left|s\left(i\right)-g\left(i\right)\right|\\ E_{L2}\left(s\right)=\frac{1}{N}\sum_{i=1}^{N}{\left(\left|s\left(i\right)-g\left(i\right)\right|\right)}^{2}=\mathrm{MAE}\left(s,g\right)\\ E_{L3}\left(s\right)=\frac{1}{N}\sum_{i=1}^{N}{\left(\left|s\left(i\right)-g\left(i\right)\right|\right)}^{3} \end{array}$$

Note that the absolute value of the difference *s*-*g* is accumulated always. If *g_i_* ≠ 0 and if there is a set *S* = {*s^j^*} of sensor signals representing different states (e.g., measured at different spatial positions), the higher-order signal energies *L*_2_ and *L*_3_ can be used to extract relevant features by relative comparison and further amplification of the signal energies. This method will be applied to CT image volume data, discussed in Section 7. The second-order signal energy is equivalent to the mean average error (MAE) if *g_i_* is a generator or reference data series, for example, retrieved by a baseline measurement. MAE is used in anomaly detectors based on, for example, autoencoders, discussed in Section 7 too.

Statistical aggregates from sensor data series are translation invariant; that is, the aggregate measure is independent of a longitudinal offset.

### 6.2. Pattern Recognition and Vision Methods

Regarding multidimensional measuring signal data, vision and pattern recognition algorithms can be used to extract suitable intermediate or damage features. Typical algorithms are kernel-based transformations (filters) aiming to intensify specific geometrical features in images. An image can be treated as a multidimensional pixel volume. There is no limitation on the dimension of such a pixel volume. The following list summarises commonly used algorithms:

- Kernel-based gradient and other edge filters for edge detection [62]:
  - Soebel filter (gradient kernel ([1 2 1][−1 0 1]));
  - Canny filter (multistage iterative, Gaussian filter kernel for noise suppression);
  - Sharr filter (([3 10 3][−1 0 1]))
- Corner detection algorithms [62]:
  - YAPE06 (Laplacian- and min-eigenvalue-based feature detector);
  - YAPE (yet another point extractor);
  - ORB (oriented and rotated BRIEF);
- Object (shape) detectors:
  - HAAR cascades object detector;
  - Brightness binary feature object detector;
- Point cloud algorithms to find point clusters forming spatially limited geometrical regions and shapes:
  - Density-based clustering (DBSCAN);
  - k-Nearest neighbor (kNN).

### 6.3. Machine Learning Methods and Models

An ML task consists of four parts:

1. Data *D* (from experiments, measurements, simulations, analytical models, numerical models) with input variables *x* and output variables *y*;
2. Labelled data: *D* = *D*(*x*,*y*);
3. Unlabelled data: *D* = *D*(*x*);
4. A model *F*(*P*), which is either a function, a directed acyclic graph (tree) whose nodes are related to input and output variables, an undirected graph whose nodes are related to data samples, or a functional directed acyclic or cyclic graph whose nodes are related to functions;
5. A parameter set *P* consisting of static and dynamic parameters, that is, *P* = *P*_static_ ∪ *P*_dyn_;
6. An algorithm that minimises the output error of |*F*(*x*)−*y*| with respect to the data or a training subset *D*_train_ ⊂ *D* by changing the dynamic (and sometimes the static) parameter variable set.

The ML methods can be classified with respect to the input and output variable category:

**ST**: Static/stationary data;
**DN**: Dynamic data (time-dependent or data series);
**NUM**: Metric and numerical data that can be interval and rationally scaled;
**CAT**: Categorical data;
**DIM**: Dimension (scalar, vector, matrix).

The analysis of time-dependent or ordered series data requires translation and rotation-invariant methods. A time series is characterised by its fundamental information content, a start and endpoint, and scaling. Only the fundamental information content are relevant signal features, not any time shift (translation), offset, or scaling (rotation) of the signal. Some methods are insensitive to translation, rotation, and scaling issues (CNN). Mostly signal transformations are applied to the raw input signal data, for example, time-to-frequency transformations.

The following methods are suitable for damage diagnostics and analysis:

**DT**: Decision trees;
**RT**: Regression trees;
**CART**: Classification and regression trees;
**ICE**: Decision trees with interval arithmetic and ε noise margin intervals:
**SVM**: Support vector machines;
**ANN**: Artificial neural networks;
**FNN**: Feed-forward neural networks;
**RNN**: Recurrent state-based neural networks with long short-term memories (LSTM);
**AE**: Autoencoders (typically implemented with FNN or RNN);
**VAE**: Variational autoencoders (typically implemented with FNN or RNN);
**CNN**: Convolutional neural networks;
**GAN**: Generative adversarial networks (with discriminators);
**PCA**: Principal component analysis, aiming to identify independent input variables, perform data alignment, and provide data reduction;
**SOM**: Self-organising maps used for feature clustering analysis and predictive classification of input data *x* (with optional output labels *y*), typically a neural network, Kohonen maps;
**kNN**: k-Nearest neighbor graph used for feature clustering;
**GMM**: Gaussian mixture model is a clustering method that preserves the geometric properties of the input space [21];
**GA**: Genetic algorithms;

Hybrid methods combine different methods either for optimising the parameter search (solving the minimisation problem), including genetic algorithms (GA), grid search (FS), or particle swarm optimisation (PSO) [20], or for fusion of methods, which are used to increase inference accuracy and robustness. The following training methods are distinguished:

**SUP**: Supervised training that requires labelled data sets {〈*x*,*y*〉};
**USP**: Unsupervised training that requires no labelled data sets {〈*x*〉};
**HYB**: Hybrid methods (e.g., combining RF with ANN [63]);
**AGL**: Agent-based learning (including reinforcement learning), typically used for hyperparameter space exploration;
**DML**: Distributed and ensemble learning [64].

Typical measuring (input) signals are:

**US**: Ultrasonic waves;
**XRAY**: X-ray;
**ACE**: Acoustic emissions;
**LIGT**: Visible or NIR light;
**T**: Temperature;
**MOI**: Moisture;
**PRS**: Pressure;
**STR**: Strain (or displacement);
**POW**: Power (light, thermal, …);
**TIME**: Time;
**POS**: Geometrical position;
**FEAT**: Any preprocessed features (e.g., of a signal).

Typical output signals are:

**DAM**: Damage class;
**POS**: Geometrical position;
**ROI**: Region-of-interest;
**CLA**: Generic classification;
**FEAT**: Coded feature vector (intermediate signal for further processing);
**TIME**: Time (e.g., lifetime prediction);
**FREQ**: Frequency;
**MAE**: Mean average error (e.g., in conjunction with AE methods).

**Figure 20:**
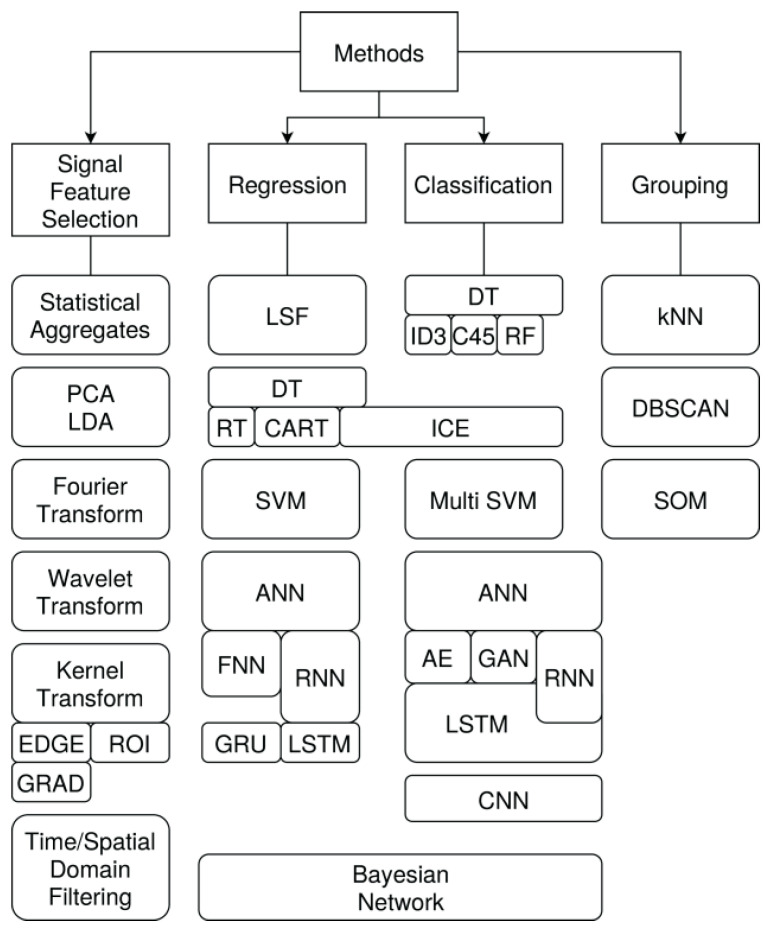
Taxonomy of damage feature extraction and prediction methods (not complete).

Some signal classes can be input and output variables too. Commonly used analysis methods are summarised in Figure 20.

#### 6.3.1. Classification

Classification aims to map measuring signal data on damage classes assuming that the signal data contain relevant information to solve the classification problem with a reasonable accuracy. That can include the binary classification {DAM, DAM}, too, see Table 1.

#### 6.3.2. Regression

Regression typically aims to predict the spatial position of a damage, an ROI, or the strength of a damage, shown in Table 2.

#### 6.3.3. Clustering

Clustering is basically used to identify groups of similar features in data sets, shown in Table 3. A cluster is a group of data rows in the data table that pose similarities. Different clusters relate commonly to different features. The features are autodetected and autoassigned. Analysing each individual cluster can deliver the dependency to the input variables *x* or a correlation to output variables *y* (e.g., a damage class). A combination of clustering with additional feature extraction methods (e.g., PCA) can improve the clustering quality and explainability.

Clustering typically maps a high-dimensional data input space on a lower-dimensional output space providing a reduced geometrical map (but not necessarily preserving input data geometries such as time).

## 7. Use Case: Non-destructive Diagnostics with Computer X-ray Tomography (CT) and Automated Damage Detection

In this section, selected experiments with X-ray CT data and FML plates are performed using and comparing classical vision-based algorithms and ML. All experiments were performed with the PSciLab software framework [67].

Basically, five methods are applied to the CT data:

1. Kernel-based transformations for edge amplification (Canny and Soebel filters) and feature marking;
2. Unsupervised training of autoencoder for anomaly detection and spatial feature marking;
3. Supervised training of state-based damage classification using LSTM networks;
4. Supervised training of state-free damage classification based on CNN;
5. Unsupervised training of self-organising (Kohonen) maps for spatial region clustering.

All five methods are applied to z-slice signals extracted from the original CT data volumes, introduced in the next sections. Method 3 was already successfully applied to GUW data for the detection of damages in CFK plates [64]. The application to CT data did not show any suitable results; therefore, the method is not described here. For details, see [64].

### 7.1. Visual Inspection

The analysis of specimens described in the next section was performed with volume and slice viewers based on the *vtk.js* visualisation framework. The specimens are multilayer plates consisting of a sandwich structure. The different layers can be viewed and separated in a cross-section volume or slice view. Damages cannot be identified clearly with volume viewers, except for spatially extended delaminations, even with intensity interval slicing. The slice view can be used to find most of the damages, such as holes, embedded pseudo defects, or cracks, but delaminations are hard to identify in slice views. The following section shows examples of volume and slice views of the different specimens under investigation. 

Depending on the resolution of the CT scan, diffraction and scattering patterns (e.g., due to simultaneous multispecimen measurements), contrast, and post-transformations, the substructure of the fibre layer can be visible and detectable or is just noisy homogeneously. Any substructure and intensity patterns in the CT images make the visual and automated damage detection more difficult.

### 7.2. Specimen and CT Image Volumes

Different specimens were tested, which are summarised in Table 4.

**Figure 21:**
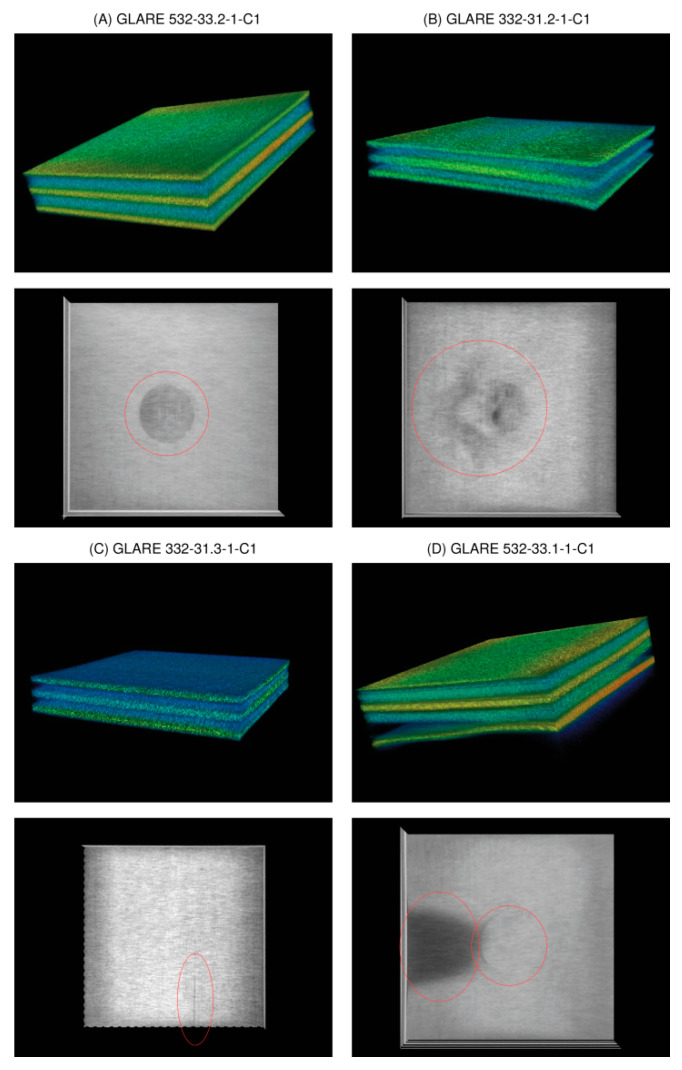
CT image volume and selected x–y slice visualisation of specimens (**A**–**D**) used in the case study (low-quality data): (**A**–**B**) with centred resin defect in the PREG layer, (**C**) fibre layer crack, (**D**) full delamination and embedded pseudo defect (foil).

**Figure 22:**
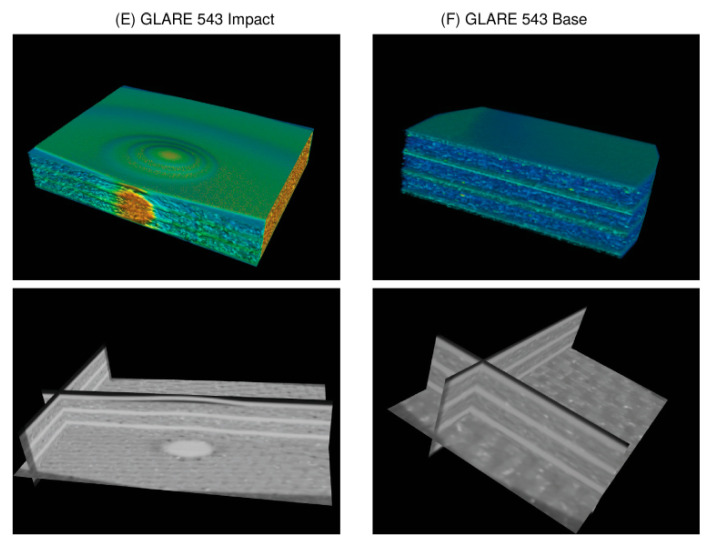
CT image volume and selected x–y slice visualisation of specimens (**E**–**F**) used in the case study (high-quality data): (**E**) with impact damage; (**F**) no damage.

Some examples of X-ray CT volume and slice visualisation are shown in Figure 21 for specimens A–D. All CT data for specimens A–D pose low spatial resolution (50 μm voxel size), and the images are of low quality due to simultaneous multispecimen measurements in the same chamber, resulting in high-intensity variations, gradients, and diffraction patterns. The CT data for specimen E pose a higher resolution (10 μm) with a higher homogeneity of the measured X-ray intensity. Figure 22 shows the volume and slice visualisation of the high-quality CT data specimens E and F. The fibre structure can be clearly identified, which is a challenge for visual and numerical damage and defect detection.

### 7.3. Geometrical Profiling and Z-Signals

CT image volumes are three-dimensional images composed of a set of two-dimensional images (image plane) reconstructed from ray imaging (e.g., X-ray or magnetic fields). The orientation of CT data volumes in space can be arbitrary, but in materials sciences and for damage diagnostics, the image plane is preferred to be either parallel to the *x*–*y* coordinate system axes (top–down view) or parallel to the x–z axes (cross-sectional view).

**Figure 23:**
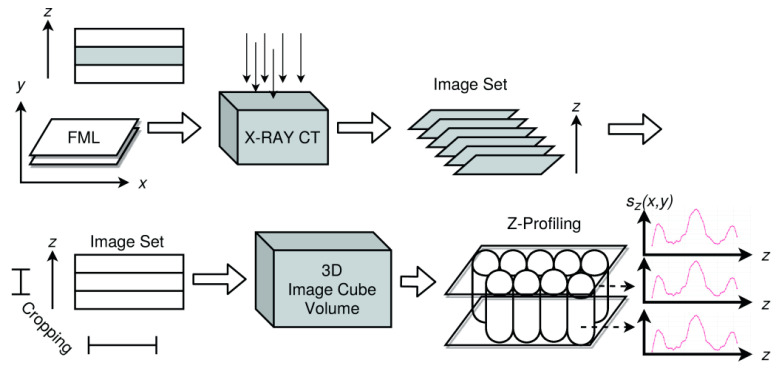
Z-slicing and transformation of 3D CT image data volumes to 2D matrix of z-signals.

Most damages and defects have a characteristic z-depth extension and pattern. Therefore, we will consider x–y plane volumes (top–down slices). Classical computer vision algorithms are applied to the entire x–y slice images, image segments, or subvolumes of the image volume. However, ML algorithms should be applied to low-dimensional data and must be translation and rotation invariant. For this purpose, we transform the x–y–z image data volume in z-signals by a z-profiling method (i.e., in a set of signals *Z* = {*z*(*x*,*y*)}) representing the top–down depth structure of specimens under test (e.g., a plate). Further transformations, such as averaging, will reduce a geometrical z-slice to a single scalar depth-resolved z-signal (a data series) at a specific centre position (*x*,*y*), shown in Figure 23.

It is assumed that single z-profiles contain damage features. For example, delaminations will stretch the signal significantly in a region (*z*_1_,*z*_2_), typically with an additional signal distortion changing the spectral features of the z-signal too. Cracks will change the z-signal with respect to geometrical and spectral features as well, depending on the measuring technique. A crack typically changes the density-material distribution and should result in an intensity change in X-ray images. Impurities (additional material (e.g., dust, fibre splinter, fluids)) will also have an impact on the z-signal but depending on the orientation and thickness of the impurity, only on a small segment of the z-signal, thus making the detection difficult.

The analysis of z-signals will not always deliver damage features immediately, but they can be used to create a two-dimensional feature marking applied finally to geometrical analysis methods, such as, point clustering and object recognition based on point clouds.

The Z-volume is computed from the original (reconstructed) X-ray CT data. The volume *V_im_* has *R* rows, *C* columns in each image x–y plane, and *D* images. Each z-slice is orthogonal to the x–y plane orientated parallel to the specimen surface (in this case, the plate surface). Rotation of the image volume can be required to achieve this alignment. 

To reduce the data size and to apply smoothing and denoising of the raw image data, a z-slice vector is computed from a region of single neighbouring z-pixels by using kernel-based transformations (e.g., averaging), that is, by the fusion of single-pixel slices of the original volume *V* at a specific position (*x*,*y*) with a radius *r*. The z-slice vector represents a cylinder or sub-cube of the entire image volume along the *z*-axis.

(3) $$\begin{array}{l} V=V\left(x,y,z\right)\in {\mathbb{R}}^{R\times C\times D}\\ s_{z}\left(k,l\right)=\frac{1}{{\left(1+2r\right)}^{2}}\sum_{i=k-r,j=l-r}^{i=k+r,j=l+r}V\left(i,j,*\right),\forall k\in \left\{r..C-r\right\},l\in \left\{r..R-r\right\} \end{array}$$

### 7.4. Signal Features

The main expected signal features are geometrical and intensity variations of the z-signal due to damages, such as delaminations, bonding defects, and impurities by embedded pseudo defects. 

#### 7.4.1. Geometrical Features

Some of the proposed analysis methods deliver intermediate feature markers, and some directly deliver a damage feature marking. Intermediate and direct damage feature images can finally be processed by geometric shape recognition and fitting. For example, cracks will pose some kind of line or line segment shape, delaminations are large rectangular or triangular shapes, and local debonding or embedded impurities can pose circular or elliptic shapes.

#### 7.4.2. Z-Signal Energy and Energy Maps

The z-signal energy of all postprocessed CT data volumes was computed to compare the signal energy feature map with other feature marking approaches. The constant base-line *g* = *s*_0_ of the signal is the average level of the entire image volume. Most spatially extended defects and damages can be detected by the signal energy feature map. To compare energy feature maps, the signal-to-noise ratio (SNR) of damage regions to background noise can be computed.

(4) $$\begin{array}{l} E{\left(x,y\right)}_{L1}=\frac{1}{N}\sum_{i=1}^{N}\left|s\left(x,y,i\right)-s_{0}\right|\\ E{\left(x,y\right)}_{L2}=\frac{1}{N}\sum_{i=1}^{N}{\left(\left|s\left(x,y,i\right)-s_{0}\right|\right)}^{2}\\ E{\left(x,y\right)}_{L3}=\frac{1}{N}\sum_{i=1}^{N}{\left(\left|s\left(x,y,i\right)-s_{0}\right|\right)}^{3}\\ s_{0}=\frac{1}{W\cdot H\cdot N}\sum_{x=1}^{W}\sum_{y=1}^{H}\sum_{z=1}^{N}s_{x,y,z}\\ SNR=\frac{\mu_{s}-\mu_{n}}{\sigma_{n}}\\ \mu_{s}=\frac{1}{n}\sum_{i,j\in B_{s}}E\left(i,j\right)\\ \mu_{n}=\frac{1}{n}\sum_{i,j\in B_{n}}E\left(i,j\right)\\ \sigma_{n}=\sqrt{\frac{1}{n-1}\sum_{i,j\in B_{n}}{\left(E\left(i,j\right)-\mu_{n}\right)}^{2}} \end{array}$$

**Figure 24:**
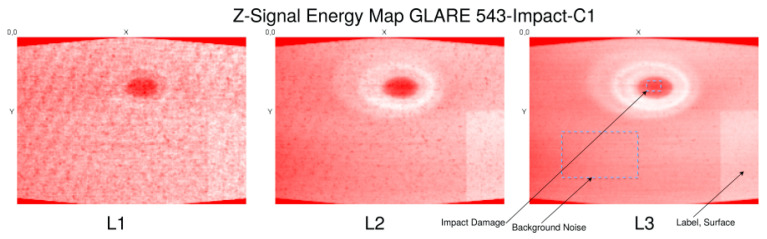
Z-signal energy map (L1, L2, L3 norms) of X-ray CT data of specimen E.

The z-signal energy map for specimen E is shown in Figure 24 for different norms (L1, L2, L3). The higher the norm, the lower the background noise of the PREG layers, and the damages and a label glued on the top of the specimen surface are amplified. Although the average SNR for L1, L2, and L3 of the impact damage (circular area) relative to the background (selected rectangular area without damage) is decreasing from 5.4 to 4.6 to 3.2, the application of a Soebel or Canny edge detection filter [62] to isolate and extract damage patterns will be more robust with less false predictions due to the smoother L3 map.

#### 7.4.3. Signal Transformations

Typical signal transformations that can be applied to the z-signal data are:

1. Gradient or deviation of the input signal;
2. Frequency transformation (DFT/FFT);
3. Discrete wavelet transformation (DWT);
4. High-pass, low-pass, or band-pass filters.

### 7.5. Anomaly Detector with an Autoencoder

An anomaly detector is an intermediate feature marker. The features can be related to desired damage features. An anomaly detector can be based on any ML model that is capable of reducing an input vector *x* to an output vector χ, representing *x* as a code. For example, transforming a signal from the time into the frequency domain is a coding too. Decoding can reconstruct the original input signal. However, in contrast to reversible coding, an anomaly detector should rely on irreversible coding that cannot reconstruct the original signal exactly, but close enough to minimise the error |s*−*s*| of the reconstructed signal. Autoencoders (AE) are typical models that are capable of coding an input variable *x* (typically a vector or a data series) on a reduced code χ with a model function *C*(*x*):*x* → χ. After decoding with an inverse function *D*(χ):χ → x*, *x* is reconstructed with a small error ε = |x*−*x*| ~ 0. There is the assumption that the code *z* represents the relevant features of the input *x*, but nothing more. If there is a modification of the input *x* due to an external effect not present in the original input (e.g., caused by damage changing *x* in any way), the AE is not able to reconstruct *x*, and the error ε ≠ 0 is increased, marking this input as an anomaly sample.

**Figure 25:**
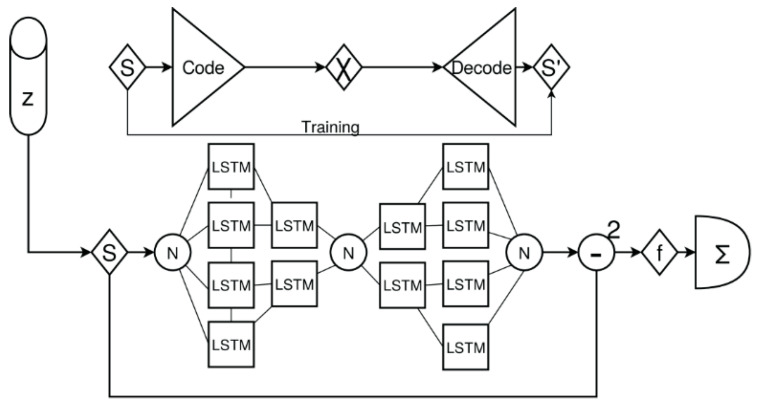
Signal anomaly detector based on a sequential AE using state-based LSTM cells with coding and a decoding stage. The z-signal data series is passed to the AE sequentially, and the error is accumulated.

The basic architecture of an AE-based anomaly detector is shown in Figure 25. Here, it is assumed that the input signal *s*(*z*) is a data series that is applied sequentially to the AE. The mean average error (MAE) is accumulated. An artificial neural network (ANN) with a combination of pure functional neurons with a sigmoid transfer function and state-based long short-term memory (LSTM) cells is used. The LSTM cells compose the coder and decoder stages. During unsupervised training using a classical gradient descent backpropagation, the error |s*(*z*)-*s*(*z*)| is minimised by updating the dynamic parameter of the network (weights of edges, bias, gates). Gaussian noise is added to the input signal to prevent learning the identity function, which is a typical issue in AE training and oversized AE networks.

**Figure 26:**
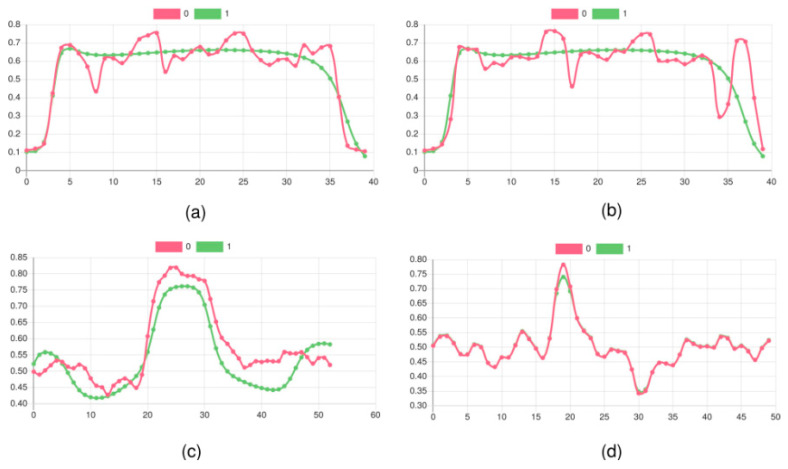
Original (0) and AE reconstructed (1) z-profile signals: (**a**) specimen E (damage-free region); (**b**) specimen E nearby impact damage; (**c**) specimen A, absolute z-signal; (**d**) specimen A, deviation of z-signal.

Experiments were carried out with specimens A, B, E, and F (see Table 4). The training was performed with selected x–y segments considered as a baseline structure. Specimens A and B were partially damaged by preparation with some sign of delamination. A true baseline does not exist. Specimen E with the impact damage poses some smaller edge areas featuring an unmodified baseline structure. The AE typically learned the average z-signal in the training region. Due to the more complex layer structure of E/F and the proper start and end image alignment, the AE learned basically an unstructured profile related to the thickness of the plate (see Figure 26a). The layer structure of specimens A/B is simpler with a higher contrast. Therefore, the AE learned the averaged X-ray intensity profile (see Figure 26b). A second experiment was performed using specimens E and F. The training was performed with the baseline specimen F, and the prediction was performed with data from E. Finally, absolute z-signals and deviation (gradient) of z-signals were tested and compared. The gradient z-signal can be reconstructed with high accuracy, but without a benefit for damage feature marking. Different symmetric architectures were evaluated, with one neuron at the input and the output and one or two LSTM layers (with and without inner layer memory-to-memory connections) in the coder and decoder stage. Suitable AE layer structures were identified with (1,15,3,2,3,15,1) and (1,9,3,2,3,9,1) layer structures (with node types (N,LSTM,LSTM,N,LSTM,LSTM,N).

**Figure 27:**
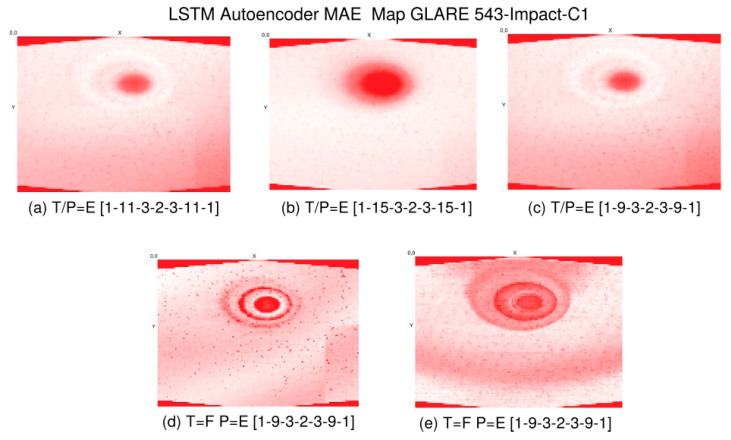
AE-MAE feature map of reconstructed AE X-ray CT data of specimen E: (**a**–**c**) training and prediction using E; (**d**,**e**) training with F, prediction with E; in brackets: AE network layer structure.

Results of AE-MAE feature maps are shown in Figure 27. The impact damage SNR of the AE-MAE-derived feature maps was for T/P = E: (a) 7.7, (b) 43.5, and (c) 7.4, and for T = F/P = E: (d) 8.8, and (e) 4.6, which is mostly higher than the SNR achieved by the simple energy maps.

Moreover, the surface label artefact was suppressed significantly by some AE. The different results from (a–c) are based on different AE network layer structures and different training with respect to randomness (in the initialisation of the network parameter) and different training epochs. AE solutions with high and low damage feature contrast will arise and vanish suddenly during the training process. The AE training is highly unstable with respect to the SNR and the core training error is no suitable measure for the quality and contrast of the post-MAE feature marking.

The training of the AE requires a large number of epochs, i.e., repeated iterations over the z-signal training data set. In this case, the training set consisted of about 500 z-slice signals randomly chosen from the entire training region. Typically, 1000–10,000 epochs with a low learning rate of about 0.01 were required. Multiple models were trained in parallel (distinguished by their different random initialisation of the network parameters), and the best models with respect to SNR were selected.

The AE-based anomaly detector is still not suitable to detect damages and isolate the spatial extension clearly, but in can be used to identify Regions-of-interest (ROI), which can be combined and further investigated by other methods (following) and to improve measurements by a zooming approach.

### 7.6. Convolutional Neural Network Classifier

**Figure 28:**
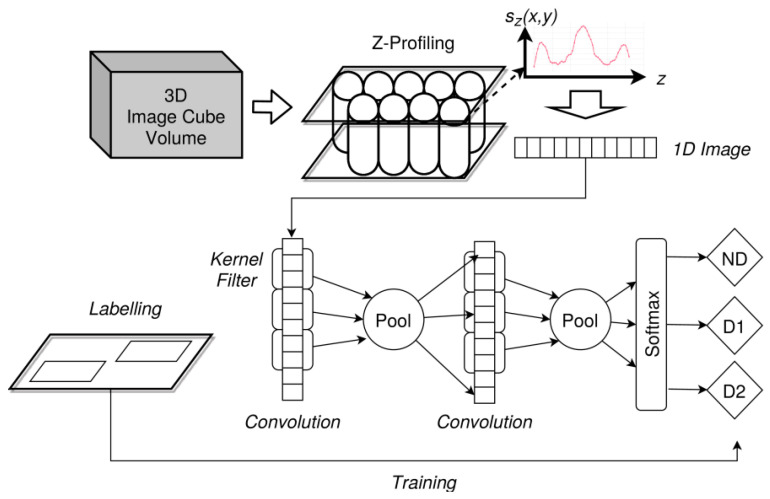
Z-profile signals as 1D images as input for a CNN damage classifier (ND: no damage class; D1: damage 1; D2: damage 2; and so on).

The second architecture that is investigated in this work is a supervised trained damage classifier based still on the z-profile signals from the previous sections. A z-signal is considered here as a one-dimensional image with signal values represented by image pixels. One main issue in damage feature detection in CT image data is the unknown geometrical position, and therefore the damage feature position in a z-signal can be at any position. Furthermore, the z-signal can be shifted or cropped due to improper image transformations and measurements. Applying sliding convolutional transformations to an image provides translation and rotation-invariant feature extraction. This is the main feature of a Convolutional Neural Network (CNN) based on sliding sub-image kernel transformations (filters) and classical neurons connected to the filters. In contrast, to the unsupervised anomaly detectors based on an AE architecture, a classical CNN requires training samples with labelled damaged regions (see Figure 28).

The CNN classifier was trained and evaluated with specimen A with a set of z-signals at different x–y positions. The output of the classifier was a set consisting of two classes: {*NoDamge*, *Damage*}. After the training, the CNN model was applied to every x–y position of the CT data volume, creating a feature map image. The resin defect was chosen as the *Damage* class. Two rectangular regions in the x–y plane were chosen to compose the labelled training data set within the damaged circular region (identified by visual inspection of the X-ray CT image slices) and within an approximately unmodified region of the plate. Different CNN architectures were investigated. A suitable CNN architecture was found with the following layer structure (changes were not critical with respect to the classification accuracy), described in Definition 1:

**Definition** **1.** *Suitable CNN layer architecture*.

[

 { type: ’input’, out_sx:|sz|, out_sy:1, out_depth:1 },

 { type: ’conv’,

   sx: 5,

   sy: 1,

   filters: 8,

   stride: 1,

   pad: 2,

   activation: ’relu’ },

 { type: ’pool’, sx: 2, sy: 1, stride: 2 },

 { type: ’conv’,

   sx: 5,

   sy: 1,

   filters: 16,

   stride: 1,

   pad: 2,

   activation: ’relu’ },

 { type: ’pool’, sx: 3, sy: 1, stride: 3 },

 { type: ’softmax’, num_classes:|classes| }

 ]

**Figure 29:**
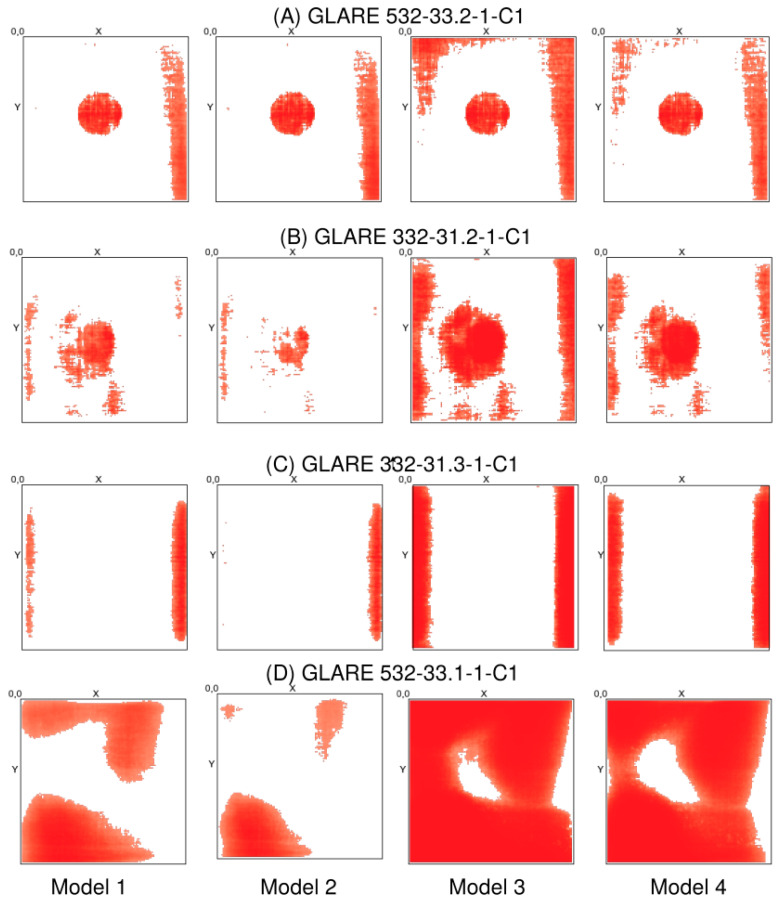
Damage feature maps retrieved from four different CNN classifiers for the specimens (**A**) (training and prediction), (**B**–**D**).

A randomly chosen training set of about 800 z-slice signals (equally partitioned with respect to the class labels and training regions) was used to train the CNN. About 200 epochs were required to achieve a low prediction error. The absolute prediction error is not relevant here since the damage classifier is applied to the entire original CT data volume. The result is a feature image with a colouring of the pixels in the x–y plane based on the classification output. Typical damage feature maps achieved by the CNN classification for specimens E and F are shown in Figure 29. The CNN was trained with specimen A. The prediction results for four different models (with respect to randomness in training) and specimen A, specimen B with a similar but blurry resin defect, and specimen C without spatially extended damages are shown. The resin damage could be clearly marked, and the damage marking for specimen B corresponded to the visual CT data inspection. For specimen C, no damage was marked, except as in all three specimens in the edge regions. This indicates a more general damage detector since the edges of the plates pose geometrical distortions, including weaker but fuzzy delamination due to cutting with a saw. The evaluation of the four models trained with the same specimen data shows a significant variation in the damage prediction accuracy and probability. Model fusion with *N* models applied to the same data can achieve a higher accuracy (spatially) and classification probability.

A completely different result is retrieved by the application of the classifier to specimen D data with a full-layer delamination damage. Here, no consistent results are achieved, and the damage feature marking does not correspond to the geometrical properties of the damage.

To summarise, the CNN damage classifier poses robustness and is sensitive to relevant damage features contained in the z-signals. The classifier is less sensitive to geometrical variations. This is demonstrated by the application of the CNN to specimen B, which is characterised by a different thickness compared with specimen A. The z-signals from B data were scaled to the length of the z-signals from A data.

### 7.7. Self-Organising Kohonen Maps

The last architecture is again an unsupervised clustering method applied to the z-profile signals. In contrast to the AE approach, a clustering method based on a neural self-organising Kohonen map (SOM) is used to group signals by nodes of the map. Each node represents some specific and characteristic (but unknown and not explainable) feature. The z-signal to SOM node mapping is used to perform a feature marking of the original CT image data (again in the *x*–*y* plane parallel to the surface of the specimen).

**Figure 30:**
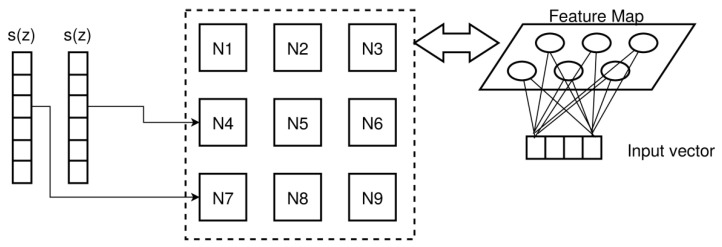
Principal concept of self-organising maps. The neural node set {n} (squares, left side) represents a feature map {f} (circles, right side).

The principal concept of a Kohonen–SOM is shown in Figure 30. A Kohonen–SOM consists of neural nodes with input edges connected to all input variables *x*, here equal to the z-signal vector of the CT data volume. The weights of the input edges determine the association of a specific input vector to one node *n_i,j_* of the network with nodes typically arranged in a two- or three-dimensional grid forming the map. The assignment of an input vector to a specific node is random; that is, each new training of a SOM adjusting the weights *w* will result in another assignment. Relevant is the binding of similar input vectors at the same node; that is, the group of vectors shares the same major feature. The specific nature and structure of the feature remain unknown. However, postanalysis of groups can extract the relevant features that can lead to the grouping (e.g., the same class of damage).

**Figure 31:**
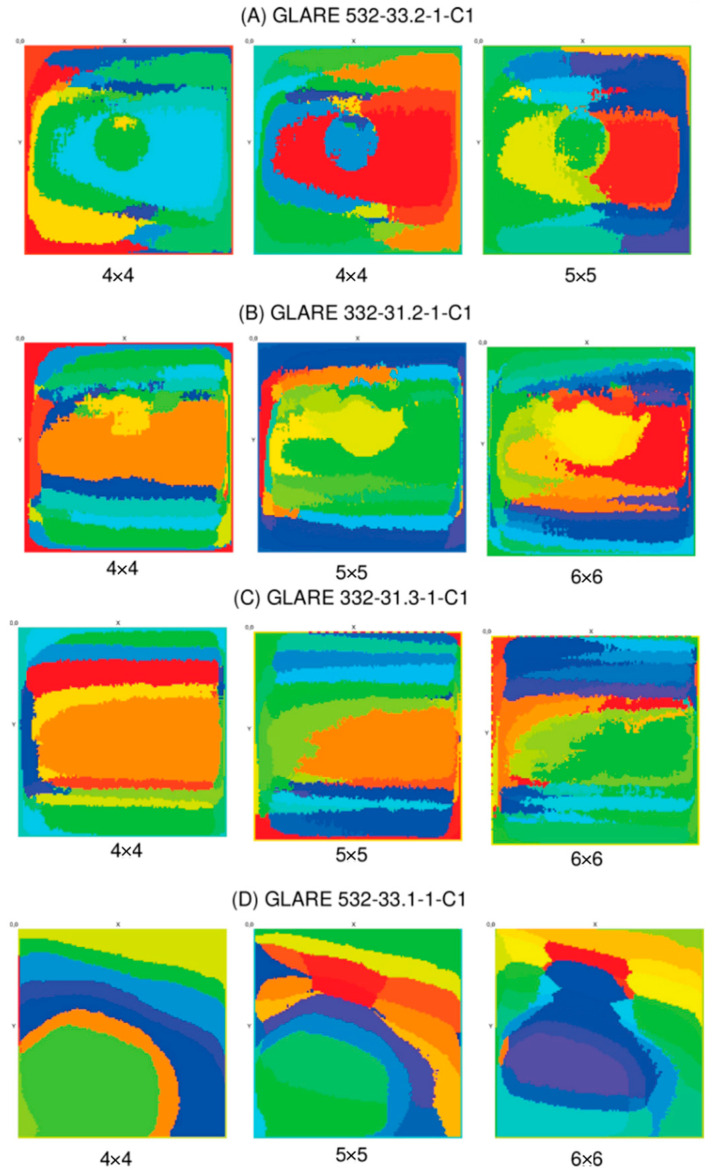
SOM feature maps of the z-signal volumes for different specimens and with different SOM network sizes (rows × columns); specimen (**A**): sharp resin washout; (**B**): fuzzy resin washout; (**C**): baseline; (**D**): large area delamination. The color code is arbitrary across different models.

SOMs were trained for each specimen data independently by using the entire z-signal data set from each specimen. After training, the mapping of each z-signal at a specific x–y position is predicted again, and a feature map based on the node set is created and visualised. Each node is represented by a discrete colour from a rainbow colour map. Pixels with the same colour mean that they are clustered on the same feature node in the SOM network. The visualisation of selected feature maps is shown in Figure 31. The feature map can vary with respect to independent training and network sizes, as shown in Figure 14 for specimen A. In the case of the sharp resin washout defect of specimen A, the feature map clearly correlates the damaged region by the assignment of the z-signals to a dedicated feature node, but this feature is also mapped on other spatial regions towards the edges of the plate (on the left side of the marked image). The major damage is marked independently of the network size.

The fuzzy resin washout defect of specimen B can be recognised by a dedicated feature mapping node. The spatial correlation compared with the visual inspection is weaker. The damage region approximates to the visually inspected region and the region identified from the CNN damage classification map if the network size is increased (up to 6 × 6 nodes). Specimen C shows no spatially limited feature regions as expected (the thin fibre crack cannot be resolved here). Finally, the feature map of specimen D with its full delamination defect corresponds well with the visual inspection, in contrast to the CNN classifier feature map. Interestingly, the spatial feature map changed significantly with the 6 × 6 networks.

The SOM method is suitable for identifying ROIs and can be used in combination with other presented methods, especially fusioned with a CNN output to strengthen the damage detection and to suppress false-positive and false-negative prediction by a spatial correlation analysis.

### 7.8. Vision Algorithms

The previous methods are only suitable for detecting extended spatial regions of damage. Fine cracks or fibre breakages are hard to find with these algorithms. The last experiment uses edge detection algorithms to isolate and amplify thin damages. Two algorithms were selected to amplify thin or line-kind damages [62]:

1. Soebel kernel-based filter (no parameters);
2. Canny multistep filter (with parameters of low and high threshold, *LT*, *HT*).

**Figure 32:**
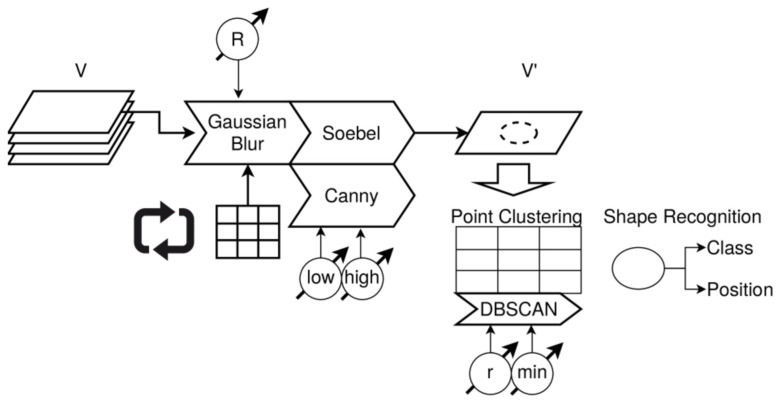
Data flow in intensity gradient and edge filter processing with final shape recognition using point cloud clustering.

**Figure 33:**
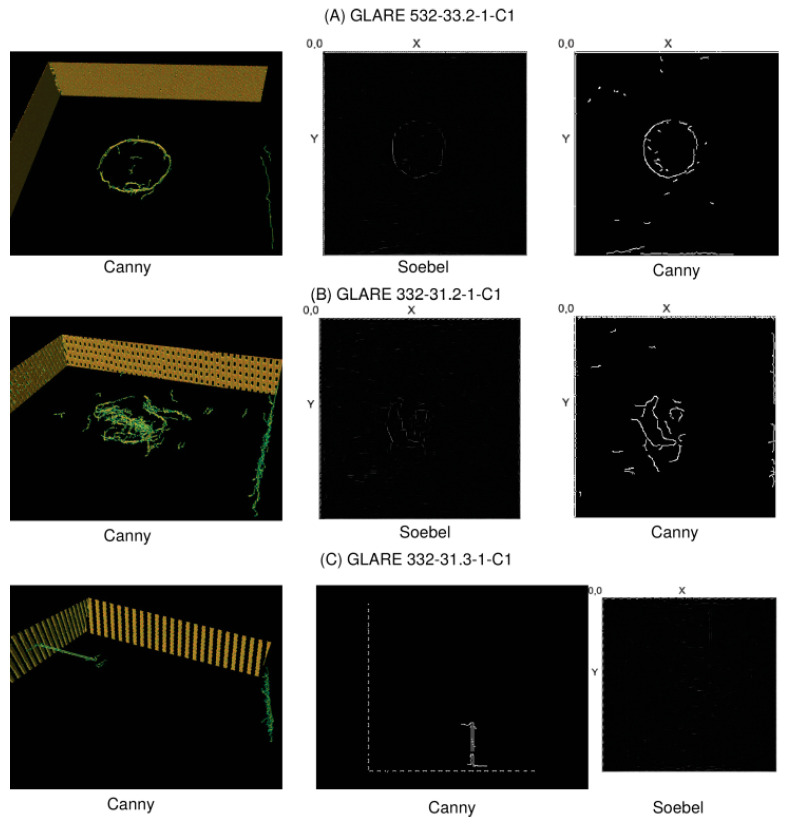
Selected results of the edge detection with the Soebel and Canny filters (volume and selected slice plots).

Here, the x–y slice images are processed separately, as shown in Figure 32. Denoising is applied first to the images by using a blur kernel filter with a blur size of about 6–8 pixels. The Soebel filter generally shows weak edge amplification of damage boundaries with a high number of short edge artefacts (noise). The Canny filter performs well and can isolate relevant damage features (resin defect and thin cracks), as shown in Figure 33. Proper choice of the low- and high-threshold parameters is critical and affects the noise and feature marking too, but typically in an opposite result. All values below *LT* are discarded, all edges with values above *HT* are selected, and potential edges with values between *LT* and *HT* are handled differently (points can create edges or not).

After edge amplification, a density-based point clustering (DBSCAN) can be applied to identify closed and dense regions, determining their centre position in the x–y plane, and finally, a shape recognition can be applied to identify typical damage shapes. This process can be ambiguous, and therefore, additional methods (such as SOM clustering) for correlation tests are required to discriminate damage features properly.

### 7.9. Summary

Detecting and discriminating different damages in composite materials from a wide range of possible damage patterns using X-ray tomography data is a challenge. Different image analysis and damage detection algorithms were introduced. Every single method is not suitable for stand-alone automated damage detection. They perform damage candidate highlighting and amplification (feature marking rather than damage detection) and can be used primarily for ROI identification. It is assumed that a fusion of different algorithms can be used for fully automated damage detection without human expert interaction. The aim of the use-case study was to highlight the challenges and limitations of even modern ML algorithms. Even simple numerical analysis, such as signal energy map diagrams, can compete with advanced ML algorithms.

## 8. Results and Discussion

The proposed taxonomy has been made comprehensive, considering the various damage patterns occurring within the composite and other laminate structures. It addresses all the characteristics currently identified in various damage patterns to the best of the authors’ knowledge and can be used in distinguishing various damage types and classes. However, it is also evident that the resulting taxonomy could evolve further based on user perception, knowledge, and experience. The proposed taxonomy remains open to all researchers, scientists, and engineers for further extension based on their respective findings and learnings. 

The classification of impact damages could be very challenging based upon different factors, such as impact energy (e.g., low impact, medium impact, and high impact energies). This is due to the fact that these impacts would produce different damage characteristics in different specimens based on the material configuration and layup. For a suitable classification with this approach, the material configuration has to be made constant to have a comprehensive classification based on the following scheme. However, in reality, the material configuration varies to a great extent in real-world application scenarios, and it is highly unlikely for two structural components or materials to be of the same configuration. In order for them to be identical, it would involve multiple factors being identical, such as metal volume fraction, fibres and fibre properties, prepreg properties, layup sequence, and orientation of the plies, which is unrealistic as a material could have different local properties in different regions based on the specific localised purpose, for example, in a region with added stiffeners to enhance the stiffness. A classification of the damage patterns based on the constant material configuration would not lead to generic classification and, hence, would not facilitate the transferability of this classification scheme. This will make the classification limited to a particular type of material configuration and would not facilitate the main goal of the research work, that is, application of this classification for damage diagnostics. For damage diagnosis, a comprehensive damage classification is important, which is not limited to a particular type of material and is applicable and transferable to multiple material configurations. However, it would offer a detailed classification of the different damage patterns observed under different impact conditions for a specific material configuration, which could be well suited for highlighting the behaviour and impact response of the material under different impact loading conditions. The application of the clinical approach in the proposed classification enables a generic classification of the damages, which is based on the intensity of the damages rather than any impact parameters or material configurations, which makes it a more qualitative approach rather than a quantitative approach. 

Detecting and discriminating different damages in composite materials from a wide range of possible damage patterns using X-ray tomography data is also a challenge. Different image analysis and damage detection algorithms were introduced. Each single method is not suitable for fully automated damage detection. They perform damage candidate highlighting and amplification. It is assumed that a fusion of different algorithms can be used for fully automated damage detection without human expert interaction. The aim of the use-case study was to highlight the challenges and the limitations even of modern ML algorithms. Even simple numerical analysis, such as signal energy map diagrams, can compete with advanced ML algorithms.

## 9. Conclusions

This paper provided a comprehensive taxonomy of the different types of damages occurring in composite and hybrid materials along with the clear definitions of the different damage modes. This proposed taxonomy could further help the researchers in seeking the definitions of such damages. Furthermore, this taxonomy would provide another perspective in identifying and organising different damages. Altogether, this work addressed methods and algorithms for damage diagnosis in hybrid and composite materials, such as FML, and introduced a novel unified taxonomy atlas of damage patterns, measuring signals, and analysis methods. Besides formal aspects, an extended use case demonstrated damage detection in FML plates using X-ray tomography data with different data analysis techniques to amplify or detect damages.

This taxonomy aims to be general, even if it is validated and tested in the domain of FMLs using ML- and image-based methods. This taxonomy highlights the first attempts towards the segregation and integration of knowledge on different damage patterns occurring in composite and hybrid materials, different analysis methods, measuring signals, and their possible correlation with various analysis methods for automated damage diagnostics. The use of a stand-alone ML algorithm cannot facilitate a damage diagnosis; however, it is more suitable for feature marking and highlighting the damaged regions. A combination of different ML and numerical image algorithms is considered to be suitable for a fully automated damage diagnosis without the requirements of any human intervention. Simple numerical analysis, such as a signal energy map, is competent enough to compete with ML algorithms. ML algorithms can be primarily used to identify spatial ROI candidates that must be investigated in depth or by using a zooming measuring technique. The zooming approach enables iterative damage diagnostics by first creating scans with the low resolution of the entire specimen and subsequently performing high-resolution scans (time consuming) of the ROI candidates.

## 10. Outlook

On the outlook, different Glare FML specimens consisting of different metal volume fractions will be impacted using an impact apparatus to replicate the actual impact damages occurring during in-service/operation. These damages replicate delamination and various other defects, such as interlaminar debonding, metal and prepreg ply delamination, matrix cracking, and fibre breakage. This proposed taxonomy of damage patterns could also be coupled with guided ultrasonic waves, where the guided wave signals will be acquired using the SHM network, allowing the damage identification. This classification could also be used to correlate the GUW signals to the damage type, followed by a class assignment to the sensor response into different classes, which could potentially help distinguish different damage patterns. The presented classification focused on the classification of different damage patterns using a data-driven approach using ML methods. Major defects, which include delamination, interlaminar debonding, matrix cracking, and fibre breakage, could be investigated with the same approach, which would facilitate a confirmation test with the proposed taxonomy. Composite materials undergo multiple modes of failure under fatigue loading, but fibre cracking and delamination seem to be the most important modes of failure that greatly affect the health of the structure. It should also be noted that the proposed classification model could possibly indicate the same results in terms of the GUW wave patterns and sensor response for different damage patterns, such as cracks and delamination. However, these damages, such as metal and matrix cracks, are associated with delamination of the plies as these two modes of failure are usually interdependent.

As more and more data are fed into the classification model, the model could lead to better-generalised results and better accuracy in predicting the pattern of the damages. Validation tests conducted on various GLARE specimens with diverse damage patterns could reveal a better accuracy of the classification model in classifying the damages. Since it is a data-driven approach, the investigations of different composite and hybrid materials with a variety of damage patterns would facilitate the training of the classification, therefore further increasing the overall accuracy of classifying different damages. Once the data structure based on the CT measurements coupled with ML-based clustering is consolidated, the accuracy, consistency, and coherency in terms of the identification of the different damage classes will increase, leading to an integrated classification model.

As an end result, this could lead to the development of a simple and comprehensive semantic overview of information derived from ontology, which is currently missing in this domain. For example, a comprehensive set of all the damage patterns has been correlated with the CT images and GUW measurements. This would enable a possibility to represent the complete information uniformly so that it can enable a complete damage diagnosis and serve the purpose of a directory for these damage patterns and the corresponding CT images and GUW measurement signals, thus further facilitating the non-destructive evaluation of these damages within composites.

## Figures and Tables

**Table 1:** Stationary (STAT) and dynamic (DYN) data matrix.

*x*	*y*	Training	Models/Methods
CAT	DAM, CAT	SUP	DT
1D; NUM; STAT: US, T, M, PRS, STR,	DAM, CAT	SUP	SVM, DT, FNN
(1D), 2D, 3D; NUM; STAT: US, XRAY, CT	DAM, CAT	SUP	CNN [65]
(1D), 2D, 3D; NUM; STAT: US, XRAY	DAM, CAT	USP	AE, CNN-AE, GAN [66]
1D, 2D; NUM; DYN: US	DAM, CAT	SUP	LSTM-RNN
1D, 2D, 3D; NUM; DYN: US, XRAY, ACE, CT	DAM, CAT	SUP	CNN, SVM [20]

**Table 2:** Stationary (STAT) and dynamic (DYN) data matrix.

*x*	*y*	Training	Models/Methods
1D; NUM; STAT: US, T, M, PRS, STR, FEAT	DAM, POS	SUP	SVM, FNN
(1D), 2D, 3D; NUM; STAT: US, XRAY	DAM, POS	SUP	CNN
1D, 2D; STAT: US, FEAT	DAM, POS	SUP	FNN
DIM1, DIM2; NUM; DYN: US, GUW, ACE	DAM, POS	SUP	LSTM-RNN [64]

**Table 3:** Stationary (STAT) and dynamic (DYN) data matrix.

*x*	*y*	Training	Models/Methods
1D, 2D, 3D, NUM; STAT: XRAY, US	GEN FEAT	USP	SOM, kNN [22]
FEAT: ACE	DAM FEAT	USO	kNN, GMM, SOM [21]
1D, 2D, 3D, NUM; DYN: XRAY, US, ACO	FEAT	USP	SOM [23]

**Table 4:** Specimen.

Name	Layers	Defect
A: GLARE 532-33.2-1-C1	3 Al, 2 PREG	Sharp spatially limited circular resin defect in the centre
B: GLARE 332-31.2-1-C1	3 Al, 2 PREG	Fuzzy spatially limited circular resin defect in the centre
C: GLARE 332-31.3-1-C1	3 Al, 2 PREG	Fibre layer crack
D: GLARE 532-33.1-1-C1	3 Al, 2 PREG	Full layer delamination with embedded pseudo defect
E: GLARE 543-Impact-C1	4 Al, 3 PREG	Impact damage
F: GLARE 543-Baseline-C1	4 Al, 3 PREG	No damage

## References

[1] Usman M., Britto R., Börstler J., Mendes E. (2017). Taxonomies in Software engineering: A systematic mapping study and a revised taxonomy development method. Inf. Softw. Technol..

[2] Vegas S., Juristo N., Basili V. (2009). Maturing software engineering knowledge through classifications: A case study on unit testing techniques. IEEE Trans. Softw. Eng..

[3] Vessey I., Ramesh V., Glass R.L. (2005). A unified classification system for research in the computing disciplines. Inf. Softw. Technol..

[4] Wohlin C. (2014). Writing for synthesis of evidence in empirical software engineering. Proceedings of the 8th ACM/IEEE International Symposium on Empirical Software Engineering and Measurement (ESEM).

[5] Cormack R.M. (1971). A review of Classification. J. R. Stat. Soc. Ser. A (Gen.).

[6] Difference between Classification and Taxonomy. https://classroom.synonym.com/difference-between-classification-taxonomy-10074596.html.

[7] (2010). Oxford Dictionary of English.

[8] Linnaeus C. (1758). System of Nature through the Three Kingdoms of Nature, According to Classes, Orders, Genera and Species, with Characters, Differences, Synonyms, Places.

[9] Tudge C. (2000). The Variety of Life.

[10] Kwasnik B.H. (1999). The role of classification in knowledge representation and discovery. Lib. Trends.

[11] Bloom B.S. (1956). Taxonomy of Educational Objectives, Volume 1: Cognitive Domain.

[12] Moffitt T.E. (1993). Adolescence-limited and life-course-persistent antisocial behavior: A developmental taxonomy. Psychol. Rev..

[13] Scharstein D., Szeliski R. (2002). A taxonomy and evaluation of dense two-frame stereo correspondence algorithms. Int. J. Comput. Vis..

[14] Kim S., Heo G., Zio E., Shin J., Song J. (2020). Cyber attack taxonomy for digital environment in nuclear power plants. Nucl. Eng. Technol..

[15] Polenghi A., Roda I., Macchi M., Pozzetti A. (2020). Data taxonomy to manage information and data in Maintenance Management. IFAC Pap. OnLine.

[16] Carvallo J.P., Franch X., Quer C., Torchiano M. Characterization of a taxonomy for business applications and the relationships among them. Proceedings of the International Conference on COTS-Based Software Systems, ICCBSS.

[17] Mahmoud M.A., Nasir N.R.M., Gurunathan M., Raj P., Mostafa S.A. (2021). The current state of the art in research on predictive maintenance in smart grid distribution network: Fault’s types, causes, and prediction methods—A systematic review. Energies.

[18] Oh Y., Witherell P., Lu Y., Sprock T. (2020). Nesting and scheduling problems for additive manufacturing: A taxonomy and review. Addit. Manuf..

[19] Frost R.B. (1994). A suggested taxonomy for engineering design problems. J. Eng. Des..

[20] Gui G., Pan H., Lin Z., Li Y., Yuan Z. (2017). Data-driven support vector machine with optimization techniques for structural health monitoring and damage detection. KSCE J. Civ. Eng..

[21] Muir C., Swaminathan B., Almansour A.S., Sevener K., Smith C., Presby M., Kiser J.D., Pollock T.M., Daly S. (2021). Damage mechanism identification in composites via machine learning and acoustic emission. Comput. Mater..

[22] Nick W., Asamene K., Bullock G., Esterline A., Sundaresan M. (2015). A study of machine learning techniques for detecting and classifying structural damage. Int. J. Mach. Learn. Comput..

[23] Emamian V., Kaveh M., Tewfik A.H. Robust clustering of acoustic emission signals using the kohonen network. Proceedings of the IEEE International Conference on Acoustics, Speech, and Signal Processing.

[24] Withers P.J., Bouman C., Carmignato S., Cnudde V., Grimaldi D., Hagen C.K., Maire E., Manley M., Plessis A.D., Stock S.R. (2021). X-ray computed tomography. Nat. Rev. Methods Primers.

[25] Glass R.L., Vessey I. (1995). Contemporary application-domain taxonomies. IEEE Softw..

[26] Wheaton G.R. (1968). Development of a Taxonomy of Human Performance: A Review of Classificatory Systems Relating to Tasks and Performance.

[27] Defects and Damages in Composite Materials and Structures. https://www.addcomposites.com/post/defects-and-damage-in-composite-materials-and-structures.

[28] Jollivet T., Peyrac C., Lefebvre F. (2013). Damage of composite materials. Procedia Eng..

[29] Talreja R., Singh C.V. (2012). Damage and Failure of Composite Materials.

[30] Leonard F., Shi Y., Soutis C., Withers P.J., Pinna C. Impact damage characterization of fibre metal laminates by X-ray computer tomography. Proceedings of the iCT Conference.

[31] Alderiesten R. (2019). Fatigue in Fibre Metal Laminates: The Interplay between Fatigue in Metals and Fatigue in Composites.

[32] Vlot A., Gunnink J.W. (2001). Fibre Metal Laminates—An Introduction.

[33] Alderliesten R.C., Homan J.J. (2006). Fatigue and damage tolerance issues of Glare in aircraft structures. Int. J. Fatigue.

[34] Abrate S. (1998). Impact on Composite Structures.

[35] Oterkus E., Diyaroglu C., De Meo D., Allegri G. (2016). Fracture modes, damage tolerance and failure mitigation in marine composites. Marine Applications of Advanced Fibre-Reinforced Composites.

[36] Jayaram S.H. Impingement of Environmental Factors that Defines a System on Composites Performance. Civil Engineering Portal. https://www.engineeringcivil.com/impingement-of-environmental-factors-that-defines-a-system-on-composites-performance.html.

[37] Shen C., Springer S.G. (1977). Environmental effects on the elastic moduli of composite materials. J. Compos. Mater..

[38] Shen C., Springer S.G. (1977). Effects of moisture and temperature on the tensile strength of composite materials. J. Compos. Mater..

[39] Rahman A.S., Shah C., Gupta N. (2020). Simultaneous effects of rice husk silica and silicon carbide whiskers on the mechanical properties and morphology of sodium geopolymer. J. Compos. Mater..

[40] Buxton A., Baillie C. (1994). A study of the influence of the environment on the measurement of interfacial properties of carbon fibre/epoxy resin composites. Composites.

[41] Haus J.N., Rittmeier L., Roloff T., Mikhaylenko A., Bornemann S., Sinapius M., Rauter N., Lang W., Dietzel A. (2021). Micro Oscillator as Integrable Sensor for Structure-Borne Ultrasound. Eng. Proc..

[42] Suriani M.J., Rapi H.Z., Ilyas R.A., Petrů M., Sapuan S.M. (2021). Delamination and manufacturing defects in natural fibre-reinforced hybrid composite: A Review. Polymers.

[43] Azzouz R., Allaoui S., Moulart R. (2021). Composite preforming defects: A review and a classification. Int. J. Mater. Form..

[44] Boisse P., Colmars J., Hamila N., Naouar N., Steer Q. (2018). Bending and wrinkling of composite fibre preforms and prepregs. A review and new developments in the draping simulations. Compos. Part B Eng..

[45] Dangora L.M., Mitchell C.J., Sherwood J.A. (2015). Predictive model for the detection of out-of-plane defects formed during textile-composite manufacture. Compos. Part A Appl. Sci. Manuf..

[46] Greenhalgh E.S. (2009). Failure Analysis and Fractography of Polymer Composites.

[47] Price W.A., Rice B.P., Crasto A.S., Thorp K.A. Hygrothermal aging of imide composites. Proceedings of the High Temple Workshop XV.

[48] Rice B.P., Lee C.W. Study of blister initiation and growth in a high temperature polyimide. Proceedings of the 29th International SAMPE Technical Conference, Disney’s Coronado Springs Resort.

[49] Hörrmann S., Adumitroaie A., Schagerl M. (2016). The effect of ply folds as manufacturing defect on the fatigue life of CFRP materials. Frat. Integrità Strutt..

[50] Potter K.D. Understanding the Origins of effects and Variability in Composites Manufacture. Proceedings of the International conference on composite materials (ICCM)-17.

[51] Dong C., Tsai T.C. (2010). Formation of resin-rich zones in composites processing. Adv. Mater. Res..

[52] Glinz J., Šleichrt J., Kytýř D., Ayalur-Karunakaran S., Zabler S., Kastner J., Senck S. (2021). Phase-contrast and dark-field imaging for the inspection of resin-rich areas and fibre orientation in non-crimp vacuum infusion carbon-fiber-reinforced polymers. J. Mater. Sci..

[53] Koutsonas S. (2018). Modelling race-tracking variability of resin rich zones on 90° composite 2.2 twill fibre curved plate. Compos. Sci. Technol..

[54] Haesch A., Clarkson T., Ivens J., Lomov S.V., Verpoest I., Gorbatikh L. (2015). Localization of carbon nanotubes in resin rich zones of a woven composite linked to the dispersion state. Nanocomposites.

[55] Lundström T.S., Gebart B.R., Lundemo C.Y. (1993). Void formation in RTM. J. Reinf. Plast. Compos..

[56] Lundström T.S., Gebart B.R. (1994). Influence from process parameters on void formation in resin transfer molding. Polym. Compos..

[57] Afendi M., Banks W.M., Kirkwood D. (2005). Bubble free resin for infusion process. Compos. Part A Appl. Sci. Manuf..

[58] Kang M.K., Lee W.I., Hahn H.T. (2000). Formation of microvoids during resin-transfer molding process. Compos. Sci. Technol..

[59] Park C.H., Lee W.I. (2011). Modeling void formation and unsaturated flow in liquid composite molding processes: A survey and review. J. Reinf. Plast. Compos..

[60] Mehdikhani M., Gorbatikh L., Verpoest I., Lomov S.V. (2019). Voids in fibre-reinforced polymer composites: A review on their formation, characteristics, and effects on mechanical performance. J. Compos. Mater..

[61] Chen D., Arakawa K., Xu C. (2015). Reduction of void content of vacuum-assisted resin transfer molded composites by infusion pressure control. Polym. Compos..

[62] JSFeat. https://inspirit.github.io/jsfeat.

[63] Wang S., Aggarwal C., Liu H. Using a Random Forest to Inspire a Neural Network and Improving on It. Proceedings of the 2017 SDM 2017: SIAM International Conference on Data Mining.

[64] Bosse S., Weiss D., Schmidt D. (2021). Supervised distributed multi-instance and unsupervised single-instance autoencoder machine learning for damage diagnostics with high-dimensional data—A hybrid approach and comparison study. Computers.

[65] Fuchs P., Kröger T., Garbe C.S. (2021). Defect detection in CT scans of cast aluminum parts: A machine vision perspective. Neurocomputing.

[66] Ghani M.U., Karl W.C. (2019). Fast enhanced CT metal artifact reduction using data domain deep learning. IEEE Trans. Comput. Imaging.

[67] Bosse S. (2022). PSciLab: An Unified distributed and parallel software framework for data analysis, simulation and machine learning—Design practice, software architecture, and user experience. Appl. Sci..

